# Nuclear hexokinase 2 couples hyperglycemia to MYC-driven glycolytic and stemness programs in bladder cancer

**DOI:** 10.1038/s41419-026-08714-0

**Published:** 2026-04-08

**Authors:** Shuangjie Liu, Xi Liu, Guangxu Liu, Zheyu Wang, Chengyi Li, Meng Yu, Jihang Yao, Haotian Xing, Yuyan Zhu

**Affiliations:** 1 Department of Urology, The First Affiliated Hospital of Bengbu Medical University, Bengbu, China; 2 Department of Urology, Cancer Hospital of China Medical University, Cancer Hospital of Dalian University of Technology, Liaoning Cancer Hospital & Institute, Shenyang, China; 3 Department of Urology, The First Hospital of China Medical University, Shenyang, China; 4 Department of Laboratory Animal Science, China Medical University. Key Laboratory of Transgenetic Animal Research, Shenyang, China; 5 Department of Gynecology, The First Hospital of China Medical University, Shenyang, China; 6 Department of Urology, The Fourth Hospital of China Medical University, Shenyang, China

**Keywords:** Cancer metabolism, Bladder cancer

## Abstract

Hyperglycemia is common in patients with bladder cancer and has been implicated in disease progression, yet the molecular link between a high-glucose milieu and tumor aggressiveness remains poorly defined. Here we identify a noncanonical, nuclear role of hexokinase 2 (HK2) that couples systemic hyperglycemia to MYC-driven glycolysis and stemness in bladder cancer. High glucose promotes nuclear translocation of HK2, where HK2 directly binds the central region of MYC to form a functional transcriptional complex. This HK2–MYC complex occupies the promoters of key glycolytic genes, including HK2 and lactate dehydrogenase A (LDHA), and synergistically activates their transcription, thereby enhancing glycolytic flux and upregulating stemness-associated markers such as CD44. Genetic or pharmacologic inhibition of HK2 attenuates high glucose–induced proliferation, colony formation, and glycolytic reprogramming in vitro. In mouse models, hyperglycemia accelerates tumor growth, whereas treatment with the HK2 inhibitor lonidamine mitigates tumor progression in the hyperglycemic setting. Analysis of human bladder cancer specimens reveals that HK2 expression positively correlates with MYC and LDHA levels and associates with worse patient survival, particularly in patients with hyperglycemia. Collectively, our findings uncover a metabolic–transcriptional coupling pathway in which nuclear HK2 functions as a MYC cofactor to drive glycolysis and stemness under high-glucose conditions, and they suggest that targeting HK2 may represent a rational therapeutic strategy for patients with bladder cancer and coexisting hyperglycemia or diabetes.

**Figure:**
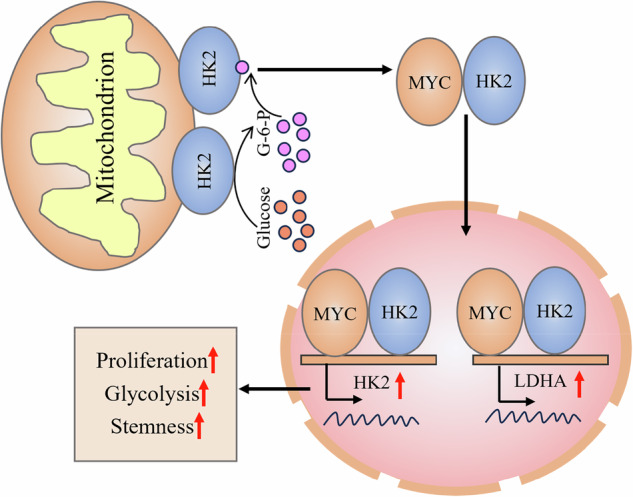
Hyperglycemia upregulates HK2 and promotes its nuclear localization in bladder cancer cells, where nuclear HK2 forms a complex with MYC to co-activate HK2 and LDHA transcription, thereby enhancing glycolysis, stemness, and tumor growth in hyperglycemic conditions.

Hyperglycemia upregulates HK2 and promotes its nuclear localization in bladder cancer cells, where nuclear HK2 forms a complex with MYC to co-activate HK2 and LDHA transcription, thereby enhancing glycolysis, stemness, and tumor growth in hyperglycemic conditions.

## Introduction

Bladder cancer is one of the most common malignancies of the urinary tract worldwide, and its high recurrence rate and resistance to therapy pose major clinical challenges [1, 2]. Bladder cancer progression, particularly invasive disease and lymph node metastasis, is driven by diverse molecular programs. Recent work highlights post-translational modification pathways regulating these processes [3, 4]. Epidemiological and clinical studies have shown that type 2 diabetes mellitus and the accompanying systemic hyperglycemia are independent risk factors for both the development and poor prognosis of bladder cancer [5, 6]. These associations suggest that a high-glucose microenvironment may play a critical role in the malignant progression of bladder cancer. In recent years, research has increasingly focused on high-glucose–driven metabolic reprogramming, namely the process by which tumor cells rewire energy metabolism to sustain rapid proliferation [7]. In bladder cancer, this metabolic reprogramming is characterized by downregulation of mitochondrial oxidative phosphorylation and enhanced glycolysis, a hallmark of the Warburg effect [8]. A high-glucose milieu can exacerbate metabolic reprogramming and alter gene regulation and therapeutic response in bladder cancer [9], but the molecular mechanisms that couple hyperglycemia to aggressive tumor programs remain incompletely defined.

Hexokinase 2 (HK2), a key rate-limiting enzyme in glycolysis, is markedly upregulated in bladder cancer [10–12]. Its transcription is regulated at multiple levels: hypoxia-inducible factor-1α (HIF-1α) and the histone demethylase JMJD1A synergistically activate the HK2 promoter [13, 14], whereas mutant p53 and MYC also transactivate HK2 to meet the metabolic demands of tumor cells [15, 16]. Recent studies have further shown that several metabolic enzymes exert noncanonical functions following nuclear translocation. For example, pyruvate kinase M2 (PKM2) translocates to the nucleus, where it phosphorylates histone H3 and acts as a transcriptional co-activator to regulate gene expression, whereas phosphoglycerate kinase 1 (PGK1) has also been reported to enter the nucleus under specific conditions and participate in transcriptional regulation [17, 18]. In this context, HK2 has likewise been shown to exert non-glycolytic functions: it activates the NF-κB pathway by phosphorylating IκBα, upregulates PD-L1 expression, and promotes immune escape [19]. However, direct experimental evidence is still lacking as to whether a high-glucose microenvironment alters the subcellular localization of HK2 and thereby drives HK2-mediated metabolic reprogramming.

As a central transcription factor, MYC regulates multiple malignant phenotypes in bladder cancer, including cell proliferation, metabolic reprogramming, and maintenance of stemness [20, 21]. MYC not only directly activates glycolytic genes such as lactate dehydrogenase A (LDHA) but also promotes PKM2 expression by upregulating the splicing factors hnRNPA1 and hnRNPA2 [22–24]. In addition, MYC directly binds to and activates the promoters of stemness-associated genes, such as OCT4, NANOG, and SOX2, thereby conferring and maintaining the self-renewal capacity of cancer stem cells (CSCs) [25, 26]. Recent studies have shown that nuclear translocation of metabolic enzymes enables their interaction with transcription factors, thereby coupling cellular metabolism to epigenetic regulation [17, 27]. For example, in ovarian cancer, hypoxia-induced ESM1 promotes SUMOylation and nuclear translocation of PKM2, which in turn phosphorylates and activates STAT3, thereby enhancing the Warburg effect [28, 29]. However, whether and how high glucose affects transcription and epigenetic remodeling by regulating interactions between metabolic enzymes and MYC in bladder cancer has yet to be systematically elucidated.

This study demonstrates that, within a high-glucose microenvironment in bladder cancer, HK2 translocates to the nucleus and forms a functional complex with the transcription factor MYC. This HK2–MYC complex directly binds to the promoters of glycolytic and stemness-associated genes and synergistically activates their transcription, thereby mechanistically coupling metabolic reprogramming to the maintenance of tumor stem cell properties. These findings not only broaden our understanding of the noncanonical functions of HK2 in bladder cancer but also reveal a novel mechanism by which a high-glucose microenvironment drives tumor malignancy through a metabolic enzyme–transcription factor axis.

## Results

### Nuclear localization of HK2 in bladder cancer cells

**Fig. 1:**
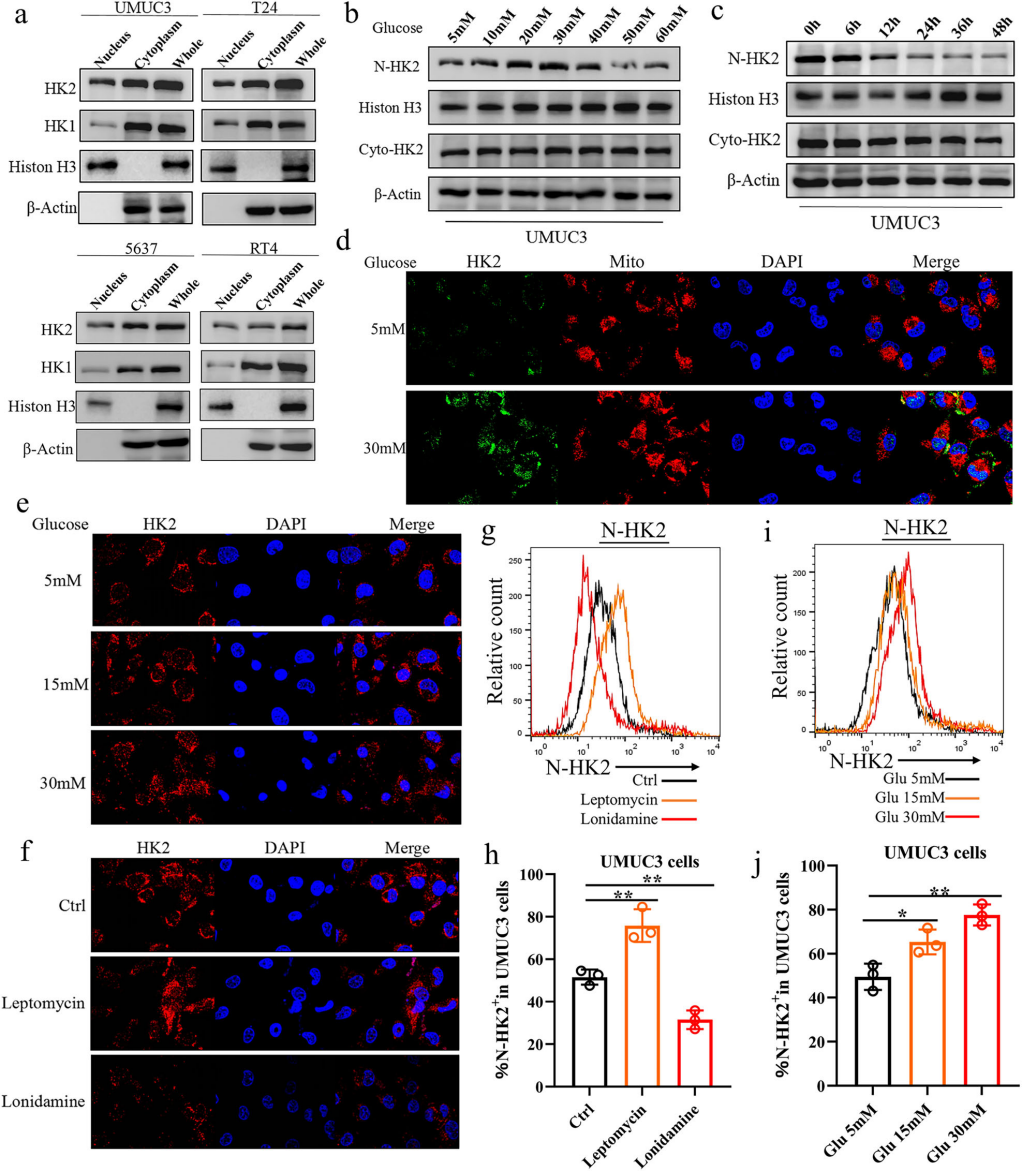
HK2 is localized to the nucleus in bladder cancer cells, and its nuclear abundance is regulated by glucose concentration. **a** Cytoplasmic, nuclear, and total levels of HK1 and HK2 in UMUC3, T24, 5637, and RT4 cells were examined by Western blotting. **b** UMUC3 cells were treated with increasing concentrations of glucose, and nuclear and cytoplasmic HK2 expression was assessed by Western blotting. **c** UMUC3 cells were exposed to a fixed concentration of glucose for the indicated times, and nuclear and cytoplasmic HK2 expression was measured by Western blotting. **d** Subcellular localization and fluorescence intensity of HK2 and mitochondria were analyzed by confocal microscopy after treatment of UMUC3 cells with different glucose concentrations. **e** Nuclear HK2 levels in UMUC3 cells treated with different glucose concentrations were examined by confocal microscopy. **f** UMUC3 cells were treated with lonidamine or leptomycin, and nuclear HK2 fluorescence was evaluated by confocal microscopy. **g**, **h** UMUC3 cells were treated as in (**e**) and nuclear HK2 levels were measured (**g**) and quantified by flow cytometry (**h**). **i**, **j** UMUC3 cells were treated as in (**f**) and nuclear HK2 levels were measured (**i**) and quantified by flow cytometry (**j**). Student’s t-test was used for statistical analysis. *P < 0.05, **P < 0.01 or ***P < 0.001 indicates a significant difference between the indicated groups.

To investigate whether members of the hexokinase family are also localized to the nucleus in bladder cancer cells, we fractionated nuclear and cytoplasmic proteins from four human bladder cancer cell lines (UMUC3, T24, 5637, and RT4) and examined the protein expression levels of HK1 and HK2. As shown in Fig. 1a, both HK1 and HK2 were detected in the nuclear fractions of all four cell lines, with HK2 being more abundant in the nucleus. Previous studies have shown that high glucose concentrations can promote nuclear translocation of HK2. Interestingly, we found that nuclear HK2 levels decreased when the extracellular glucose concentration exceeded 40 mM (Fig. 1b and Supplementary Fig. 1a). Under 40 mM glucose, HK2 levels in both the cytoplasmic and nuclear fractions gradually declined with prolonged treatment (Fig. 1c and Supplementary Fig. 1b). As shown in Fig. 1d and e, increasing glucose from 5 to 30 mM led to a progressive increase in HK2 expression and nuclear accumulation, with substantial redistribution of HK2 from mitochondria to the nucleus. Consistent with its role as a classical HK2 inhibitor, lonidamine markedly reduced HK2 protein levels. Leptomycin, a classical inhibitor of CRM1-dependent nuclear export, was used to assess HK2 nuclear export [30, 31]. Immunofluorescence staining showed that leptomycin blocked nuclear export of HK2 and increased nuclear HK2 levels in UMUC3 cells, whereas lonidamine reduced nuclear HK2 levels (Fig. 1f). Consistent with these findings, flow cytometric analysis demonstrated that increasing glucose concentrations promoted nuclear accumulation of HK2, leptomycin further enhanced its nuclear retention, and lonidamine decreased nuclear HK2 levels (Fig. 1g–j). Together, these data indicate that HK2 is localized in the nucleus of bladder cancer cells and that its nuclear localization is dynamically regulated by glucose concentration.

### HK2 and MYC form a protein complex in bladder cancer cells

**Fig. 2:**
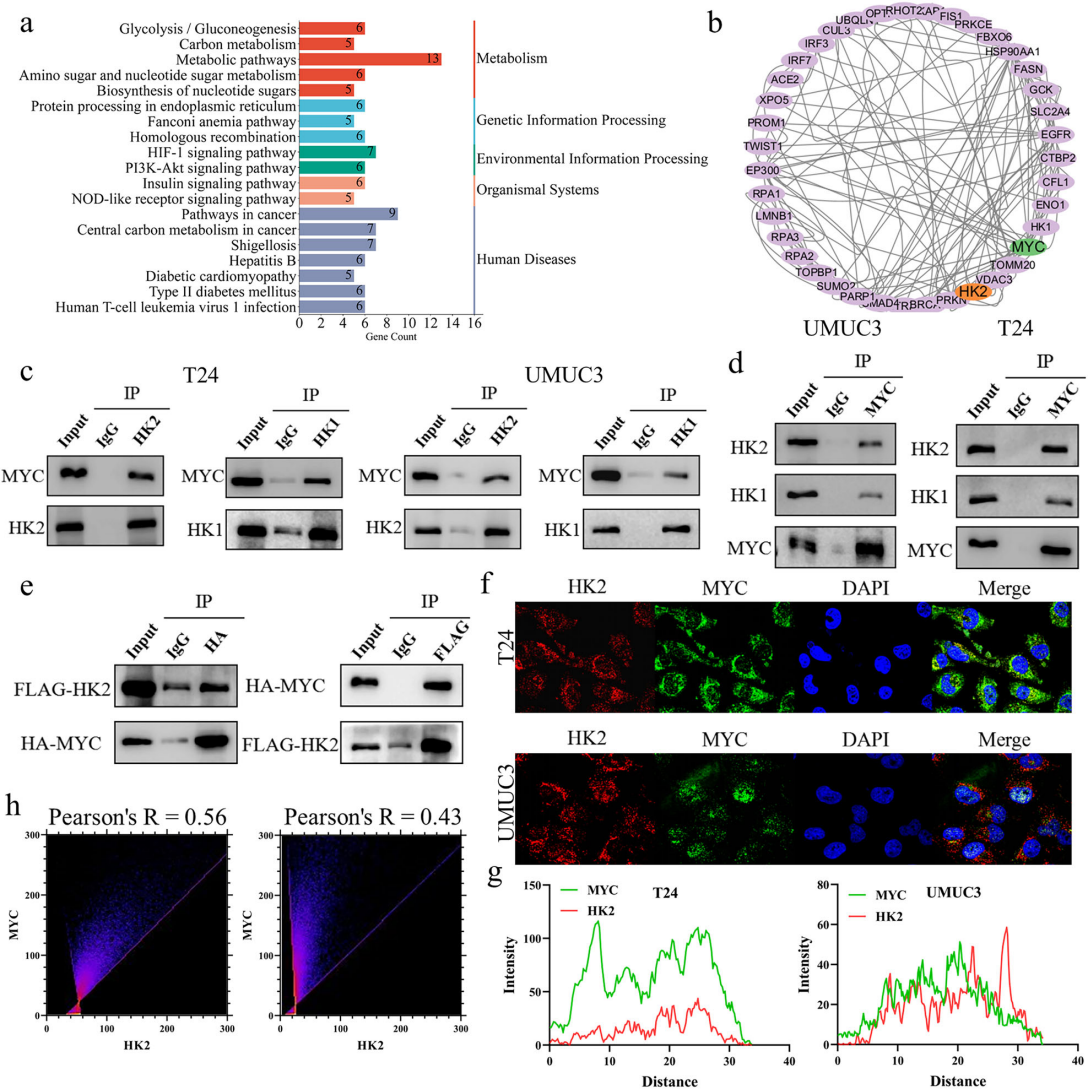
HK2 and MYC form a protein complex in bladder cancer cells. **a** KEGG pathway analysis of HK2-associated proteins retrieved from the BioGRID database. **b** HK2 protein–protein interaction network visualized using Cytoscape. **c**, **d** Co-immunoprecipitation (Co-IP) analysis of endogenous HK2 or HK1 with MYC in T24 and UMUC3 cells. **e** Co-IP analysis of the interaction between transfected FLAG-HK2 and HA-MYC in HEK293T cells. **f** Subcellular distribution and colocalization of HK2 and MYC in T24 and UMUC3 cells analyzed by confocal microscopy. **g**, **h** UMUC3 and T24 cells were prepared as in (**f**) and fluorescence colocalization (**g**) and Pearson’s correlation coefficients (**h**) were quantified.

Previous studies have shown that HK2 binds to the transcription factor MAX, which frequently heterodimerizes with MYC to regulate transcription. We therefore hypothesized that HK2 might also interact directly with MYC. Analysis of HK2-interacting proteins in mass spectrometry datasets curated in BioGRID indicated that HK2 participates in genetic information–processing pathways and suggested a potential protein–protein interaction between HK2 and MYC (Fig. 2a and b). Consistent with this prediction, co-immunoprecipitation (Co-IP) assays in the bladder cancer cell lines UMUC3 and T24 demonstrated that both HK1 and HK2 formed protein complexes with MYC (Fig. 2c and d). To further validate the interaction between HK2 and MYC, we co-transfected FLAG-HK2 and HA-MYC expression plasmids into HEK293T cells and performed co-immunoprecipitation (Co-IP) using anti-HA and anti-FLAG antibodies. These experiments confirmed that HK2 interacts with MYC (Fig. 2e). Confocal immunofluorescence microscopy revealed extensive co-localization of HK2 and MYC at the nuclear periphery and within the nucleus (Fig. 2f–h). Together, these results support the formation of an HK2–MYC protein complex in mammalian cells.

### Nuclear HK2 enhances expression of glycolytic genes

**Fig. 3:**
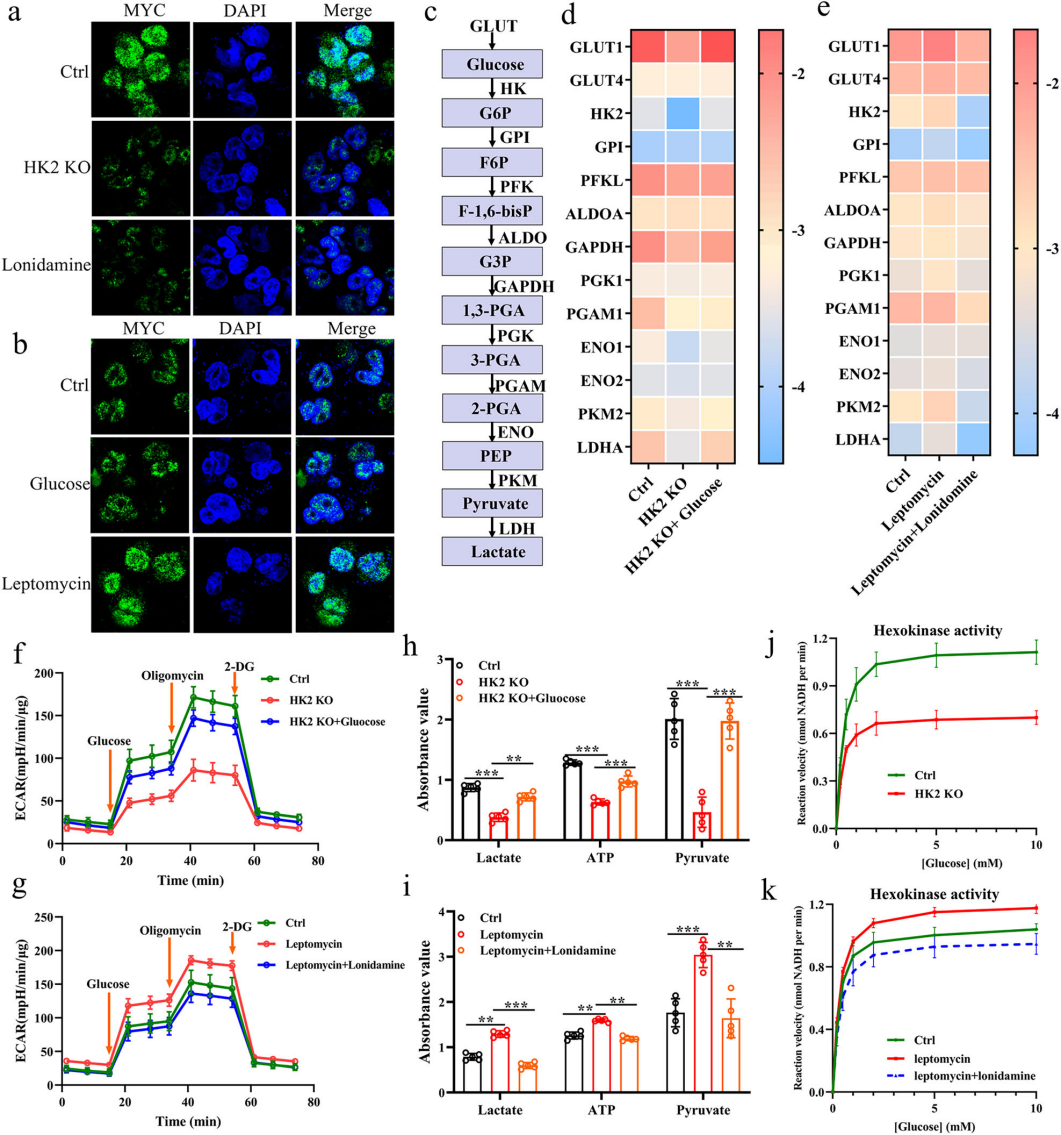
Nuclear HK2 regulates glycolytic gene expression and glycolytic capacity. **a** UMUC3 cells were subjected to HK2 knockdown or treated with lonidamine, and nuclear HK2 levels were examined by confocal microscopy. **b** UMUC3 cells were treated with high-glucose medium or leptomycin, and nuclear HK2 levels were examined by confocal microscopy. **c** Schematic representation of glycolysis-related genes and their intermediate metabolites analyzed in this study. **d** UMUC3 cells were treated as in (**a**) and the mRNA expression of glycolysis-related genes was measured by qPCR. **e** UMUC3 cells were treated as in (**b**) and the mRNA expression of glycolysis-related genes was measured by qPCR. **f** UMUC3 cells were treated as in (**a**) and the extracellular acidification rate (ECAR) was measured. **g** UMUC3 cells were treated as in (**b**), and ECAR was measured. **h** UMUC3 cells were treated as in (**a**) and lactate production, ATP levels, and glucose uptake were quantified. **i** UMUC3 cells were treated as in (**b**), and lactate production, ATP levels, and glucose uptake were quantified. **j** UMUC3 cells with HK2 knockdown were analyzed for hexokinase activity. **k** UMUC3 cells were treated as in (**b**) and hexokinase activity was measured. Student’s t-test was used for statistical analysis. *P < 0.05, **P < 0.01 or ***P < 0.001 indicates a significant difference between the indicated groups.

To investigate the functional consequences of the HK2–MYC interaction, we first examined MYC expression after modulating HK2 levels. Immunofluorescence analysis showed that knockdown or pharmacological inhibition of HK2 reduced MYC signal, whereas increased HK2 expression led to enhanced MYC accumulation (Fig. 3a and b). Consistently, HK2 knockdown in UMUC3 cells decreased the expression of glycolytic genes; notably, high-glucose treatment attenuated this suppression of glycolytic gene expression (Fig. 3c and d). Furthermore, treatment of UMUC3 cells with leptomycin, which promotes nuclear retention of HK2, increased glycolytic gene expression, whereas lonidamine attenuated the leptomycin–induced upregulation of these glycolytic genes (Fig. 3e). Similarly, the extracellular acidification rate (ECAR) in UMUC3 cells was reduced by HK2 knockdown, whereas high-glucose treatment attenuated this decrease (Fig. 3f). Leptomycin increased ECAR in UMUC3 cells, whereas lonidamine mitigated the leptomycin–induced elevation in ECAR (Fig. 3g). Consistently, HK2 knockdown reduced glycolytic lactate production, ATP levels, and glucose uptake, while high-glucose treatment partially reversed these effects (Fig. 3h). Conversely, leptomycin increased lactate production, ATP levels, and glucose uptake in UMUC3 cells, whereas lonidamine attenuated the leptomycin–dependent enhancement of these glycolytic parameters (Fig. 3i). We also observed that HK2 knockdown reduced total hexokinase activity. Conversely, leptomycin increased hexokinase activity, whereas lonidamine attenuated the leptomycin–induced increase (Fig. 3j and k). Together with the above findings, these data support a model in which HK2–MYC interaction promotes glycolytic activity in bladder cancer cells.

### Nuclear HK2 promotes stemness and proliferation of bladder cancer cells

**Fig. 4:**
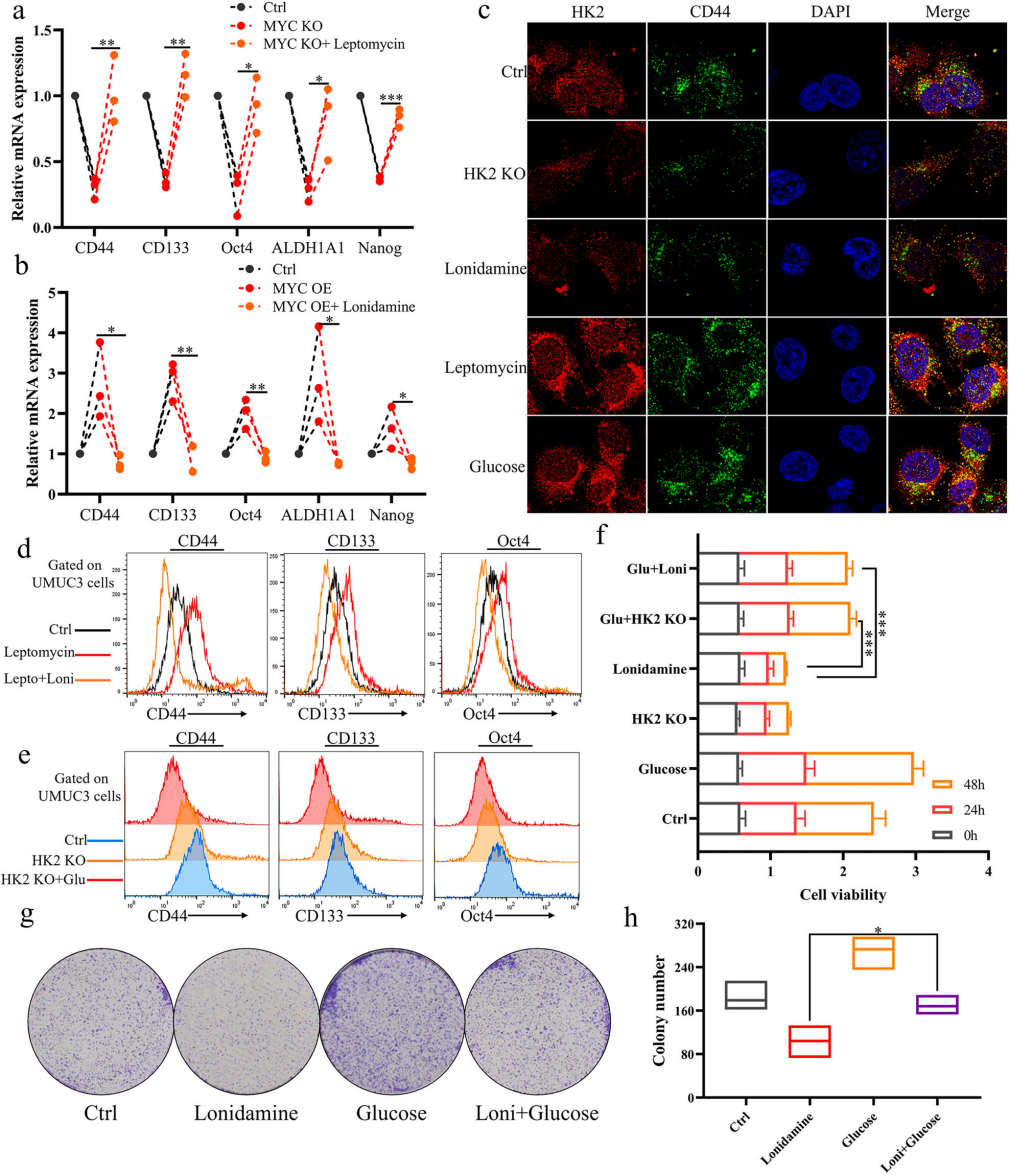
Nuclear HK2 enhances expression of stemness-related genes and promotes proliferation of bladder cancer cells. **a** UMUC3 cells were subjected to MYC knockdown with or without leptomycin treatment, and mRNA expression of stemness-related genes was measured by qPCR. **b** UMUC3 cells overexpressing MYC were treated with or without lonidamine, and mRNA expression of stemness-related genes was measured by qPCR. **c** UMUC3 cells were subjected to HK2 knockdown or treated with lonidamine, leptomycin, or high-glucose medium, and HK2 and CD44 expression were examined by confocal microscopy. **d** UMUC3 cells were treated with leptomycin with or without lonidamine, and CD44, CD133, and OCT4 protein expression was analyzed by flow cytometry. **e** UMUC3 cells were subjected to HK2 knockdown with or without lonidamine, and CD44, CD133, and OCT4 protein expression was analyzed by flow cytometry. **f** UMUC3 cells cultured in high-glucose medium were subjected to HK2 knockdown or treated with lonidamine for the indicated times, and cell viability was assessed by CCK-8 assay. **g**, **h** UMUC3 cells were treated with lonidamine with or without high-glucose conditions; cell proliferation was evaluated by colony formation assay (**g**) and colony numbers were quantified (**h**). Student’s t-test was used for statistical analysis. *P < 0.05, **P < 0.01 or ***P < 0.001 indicates a significant difference between the indicated groups.

Numerous studies have demonstrated that MYC regulates tumor cell stemness. We therefore asked whether HK2 cooperates with MYC to control stem-like properties in bladder cancer. As shown in Fig. 4a, b, MYC knockdown in UMUC3 cells decreased the expression of stemness-associated genes CD44, CD133, OCT4, ALDH1A1, and NANOG, whereas leptomycin treatment attenuated this suppression of stemness gene expression. Consistently, immunofluorescence analysis revealed that HK2 knockdown or pharmacological inhibition reduced CD44 expression, whereas increased nuclear HK2 levels enhanced CD44 expression (Fig. 4c). Flow cytometric analysis was used to quantify expression of the stemness markers CD44, CD133, and OCT4. Leptomycin increased expression of these markers in bladder cancer cells, whereas lonidamine attenuated the leptomycin–induced upregulation. Conversely, HK2 knockdown reduced expression of CD44, CD133, and OCT4, and high-glucose treatment partially reversed this suppression (Fig. 4d and e). Subsequently, we examined the effects of these treatments on UMUC3 cell viability. High-glucose treatment increased UMUC3 cell viability, whereas lonidamine or HK2 knockdown attenuated this glucose-induced effect (Fig. 4f). Consistently, high glucose promoted UMUC3 cell proliferation, while lonidamine largely abolished the pro-proliferative effect of glucose (Fig. 4g, h). Together with the above data, these findings support a model in which nuclear HK2 promotes stemness and proliferation in bladder cancer cells.

### HK2 directly binds to the central region of MYC

**Fig. 5:**
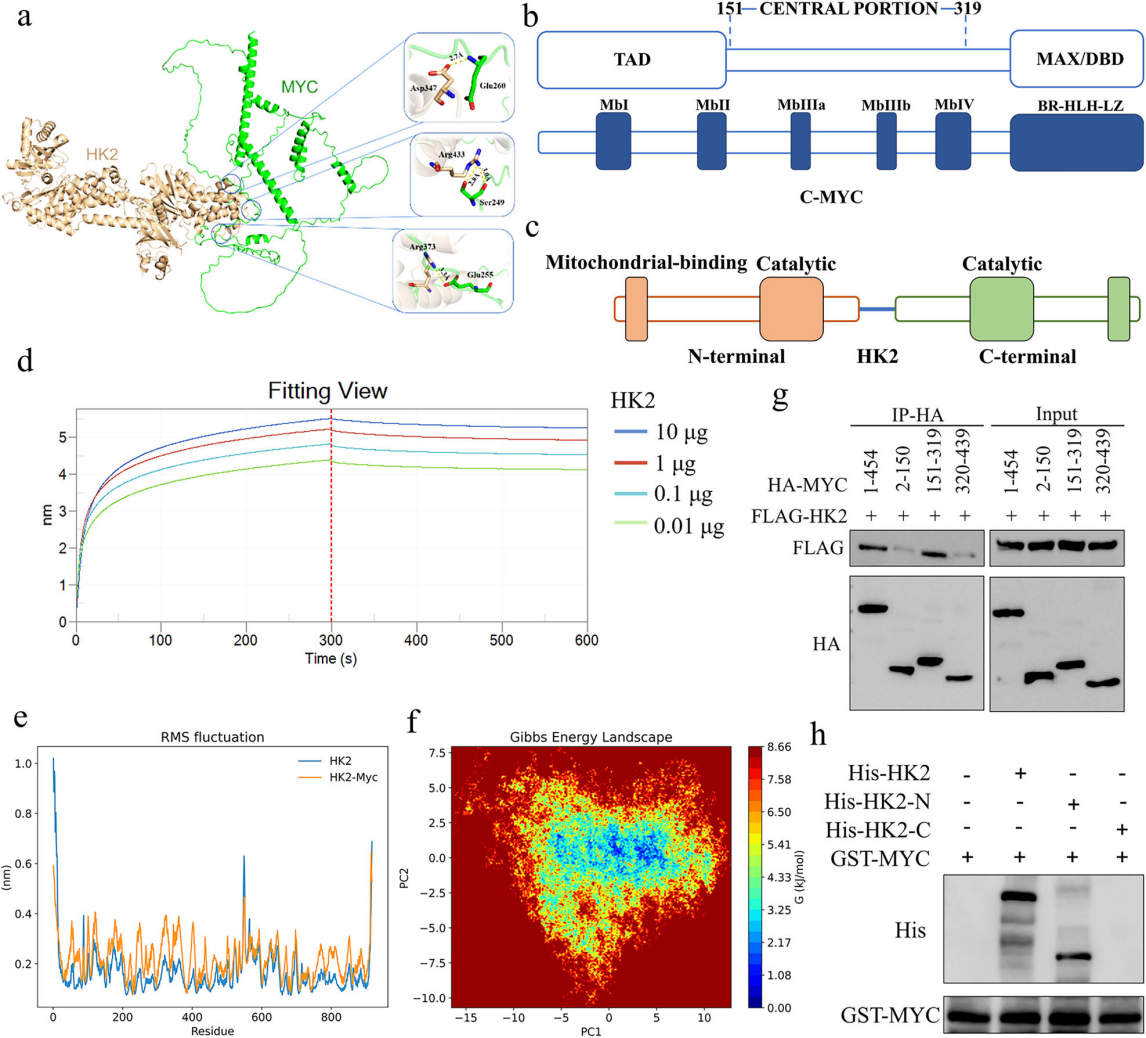
HK2 directly binds to the central region of MYC. **a** Protein–protein docking between HK2 and MYC was performed using ZDOCK. (b-c) Schematic representation of the domain organization of MYC (**b**) and HK2 (**c**). **d** Biolayer interferometry (Octet K2) analysis of HK2–MYC binding. **e** Root-mean-square deviation (RMSD) profiles of HK2 alone and the HK2–MYC complex over a 100 ns molecular dynamics simulation. **f** Gibbs free energy landscape of the HK2–MYC complex derived from a 100 ns molecular dynamics simulation. **g** Co-immunoprecipitation (Co-IP) analysis of the interaction between HK2 and different MYC domains. **h** GST pull-down analysis of the interaction between MYC and different HK2 domains.

To further characterize the binding mode between HK2 and MYC, we performed protein–protein docking using ZDOCK. As shown in Fig. 5a–c and Supplementary Fig. 2a, the N terminus of HK2 is tightly engaged with the MbIII domain of MYC, extending deeply into the MbIII cavity, whereas the predicted binding interface between HK1 and MYC is more dispersed and appears weaker. To experimentally validate the HK2–MYC interaction, we next used biolayer interferometry (BLI) to measure the binding affinity between purified HK2 and MYC. Consistent with the docking results, HK2 bound directly to MYC with high affinity (Fig. 5d). Furthermore, molecular dynamics (MD) simulations were carried out to probe the stability and conformational dynamics of the HK2–MYC complex. Root mean square fluctuation (RMSF) analysis showed that HK2 in complex with MYC exhibited larger backbone fluctuations than free HK2, indicating increased conformational flexibility upon MYC binding (Fig. 5e). Furthermore, the Gibbs free energy landscape indicated that the HK2–MYC complex predominantly sampled a stable low-energy conformational state during the molecular dynamics simulations (Fig. 5f). To determine which MYC domains mediate binding to HK2, HA-tagged MYC truncation constructs were co-transfected with FLAG-HK2 into HEK293T cells. Co-immunoprecipitation (Co-IP) assays showed that the central region of MYC was responsible for binding to HK2 (Fig. 5g). Similarly, FLAG-tagged N-terminal or C-terminal fragments of HK2 were co-transfected with HA-MYC into HEK293T cells, and Co-IP analysis revealed that the N-terminal region of HK2 mediated the interaction with MYC (Supplementary Fig. 2b). In addition, purified bacterially expressed GST-MYC pulled down full-length His-HK2 and the His-HK2 N-terminal fragment, but not the C-terminal fragment (Fig. 5h). Together, these results demonstrate that HK2 directly binds to the central region of MYC via its N-terminal domain.

### The HK2–MYC complex is enriched at the HK2 and LDHA promoters to regulate their transcription

**Fig. 6:**
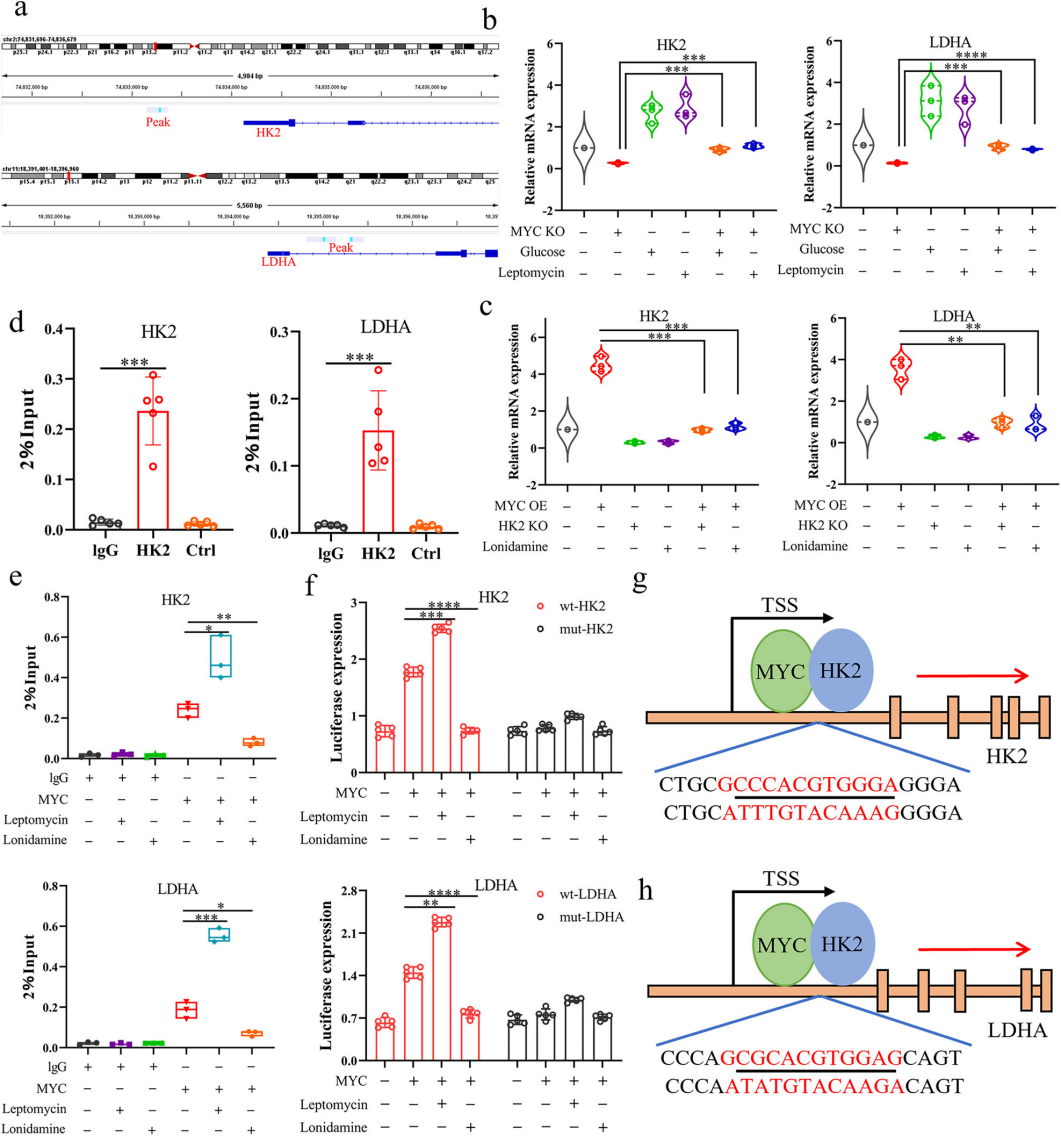
The HK2–MYC transcriptional complex binds the HK2 and LDHA promoters to regulate their transcription. **a** Enrichment of nuclear HK2 at the HK2 and LDHA promoter regions was assessed by analysis of ChIP–seq data (GSM5354447). **b** UMUC3 cells subjected to MYC knockdown were cultured in high-glucose medium or treated with leptomycin, and HK2 and LDHA mRNA expression was measured by qPCR. **c** UMUC3 cells overexpressing MYC were subjected to HK2 knockdown or treated with lonidamine, and HK2 and LDHA mRNA expression was measured by qPCR. **d** Enrichment of HK2 at the HK2 and LDHA promoters in UMUC3 cells was analyzed by ChIP assay. **e** UMUC3 cells were treated with leptomycin or lonidamine, and MYC enrichment at the HK2 and LDHA promoters was analyzed by ChIP assay. **f** Luciferase reporter assay: UMUC3 cells were transfected with wild-type or MYC-binding-site–mutant HK2 or LDHA promoter–luciferase constructs. After transfection, cells overexpressing MYC were treated with or without leptomycin or lonidamine, and luciferase activity was measured. **g**, **h** Schematic representation of HK2–MYC complex binding sites within the HK2 (**g**) and LDHA (**h**) genomic loci. Student’s t-test was used for statistical analysis. *P < 0.05, **P < 0.01 or ***P < 0.001 indicates a significant difference between the indicated groups.

As a transcription factor, MYC has been shown to occupy the promoter regions of HK2 and LDHA and regulate their transcription. Because HK2 and MYC interact in the nucleus, we hypothesized that HK2 might also participate in the transcriptional regulation of glycolytic genes. By analyzing public ChIP-seq data (GSM5354447) from the GEO database, we found that nuclear HK2 was enriched at the promoter regions of the HK2 and LDHA genes (Fig. 6a). Moreover, MYC knockdown decreased HK2 and LDHA expression, whereas MYC overexpression increased their expression. Notably, high-glucose or leptomycin treatment attenuated MYC-dependent induction of HK2 and LDHA transcript levels (Fig. 6b,c). ChIP assays showed that HK2 was enriched at the promoter regions of the HK2 and LDHA genes (Fig. 6d). As shown in Fig. 6e, leptomycin increased MYC occupancy at the HK2 and LDHA promoters, whereas lonidamine reduced MYC enrichment at these promoters. To further assess the requirement of MYC binding for HK2–MYC–dependent transcription of HK2 and LDHA, we mutated the MYC-binding motifs within the promoter regions of these genes. Under these conditions, luciferase reporter assays showed that leptomycin and lonidamine no longer altered MYC-mediated transcriptional activity of the HK2 and LDHA promoters (Fig. 6f–h). Together, these results indicate that nuclear HK2 is enriched at the HK2 and LDHA promoters and cooperates with MYC to regulate their transcription.

### Hyperglycemia promotes nuclear HK2 accumulation and bladder cancer growth in vivo

**Fig. 7:**
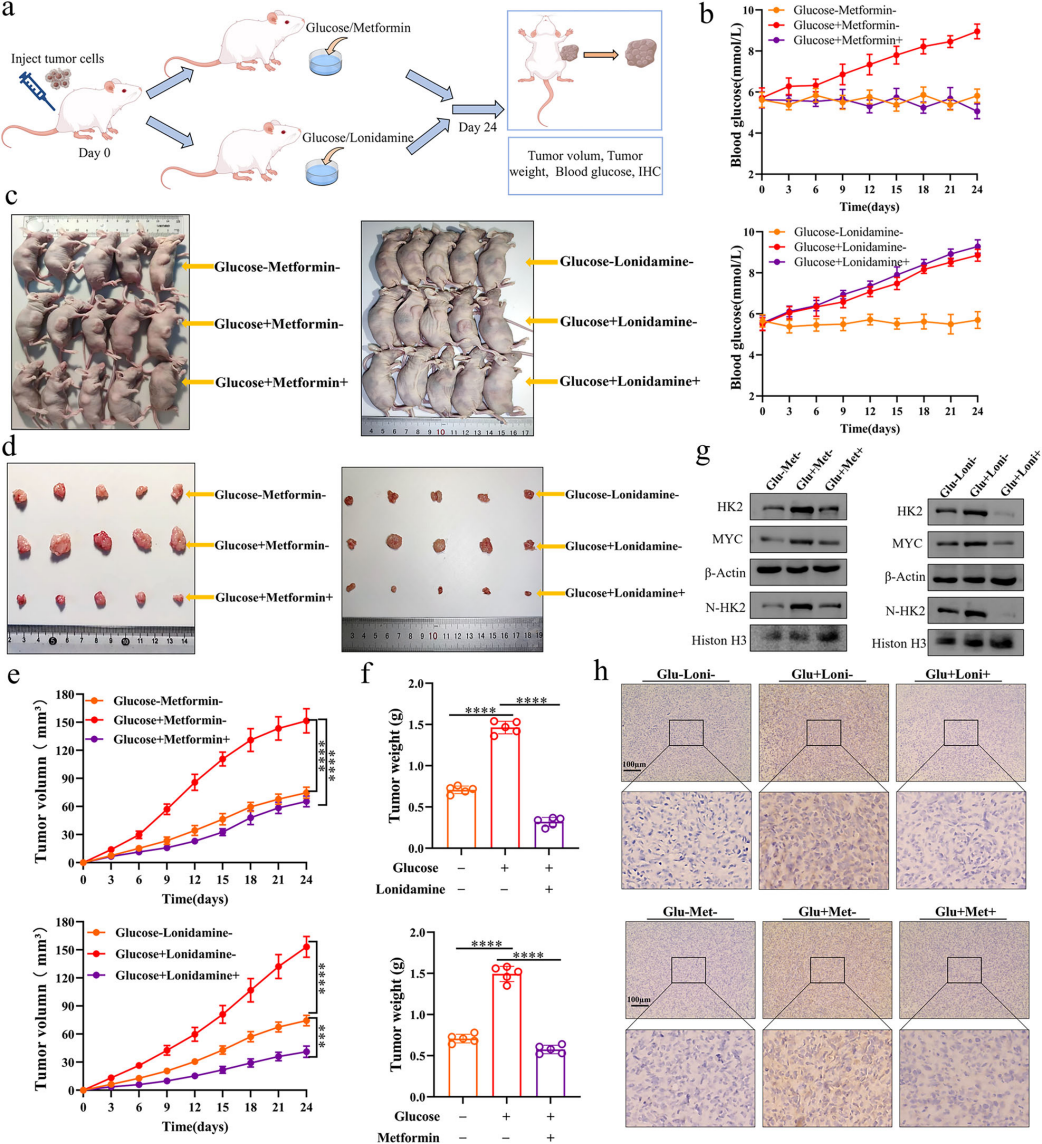
Hyperglycemia induces HK2 upregulation and promotes bladder cancer growth in vivo. **a** Schematic of the subcutaneous bladder cancer xenograft model in nude mice and treatment regimens (hyperglycemia, metformin or lonidamine). **b** Blood glucose levels in mice subjected to the indicated treatments. Representative images of nude mice (**c**) and excised tumors (**d**). **e**, **f** Tumor volume (**e**) and tumor weight (**f**) were quantified. **g** Western blot analysis of whole-cell lysates from xenograft tumors for the indicated proteins. **h** HK2 expression in xenograft tumors was examined by immunohistochemistry. Student’s t-test was used for statistical analysis. *P < 0.05, **P < 0.01 or ***P < 0.001 indicates a significant difference between the indicated groups.

To further investigate the roles of glucose and nuclear HK2 in bladder cancer, we established a subcutaneous xenograft model of bladder cancer in nude mice. Mice were fed a high-sugar diet to induce hyperglycemia and then treated with metformin or lonidamine. As shown in Fig. 7a, b, the high-sugar diet increased blood glucose levels, whereas metformin markedly reduced this effect; in contrast, lonidamine did not significantly alter blood glucose levels. Nevertheless, elevated blood glucose levels accelerated tumor growth, and both metformin and lonidamine significantly inhibited this hyperglycemia-driven tumor progression and slowed tumor growth (Fig. 7c–f). Western blot analysis of xenograft tumor tissues showed that both metformin and lonidamine treatment reduced high-glucose–induced expression of cytoplasmic HK2, nuclear HK2, and MYC (Fig. 7g). Consistently, immunohistochemical staining demonstrated that metformin and lonidamine attenuated high-glucose–driven upregulation of HK2 protein (Fig. 7h). Together, these findings suggest that either lowering blood glucose levels or inhibiting HK2 can suppress bladder cancer growth in vivo.

### HK2 is highly expressed in bladder cancer and positively correlated with MYC

**Fig. 8:**
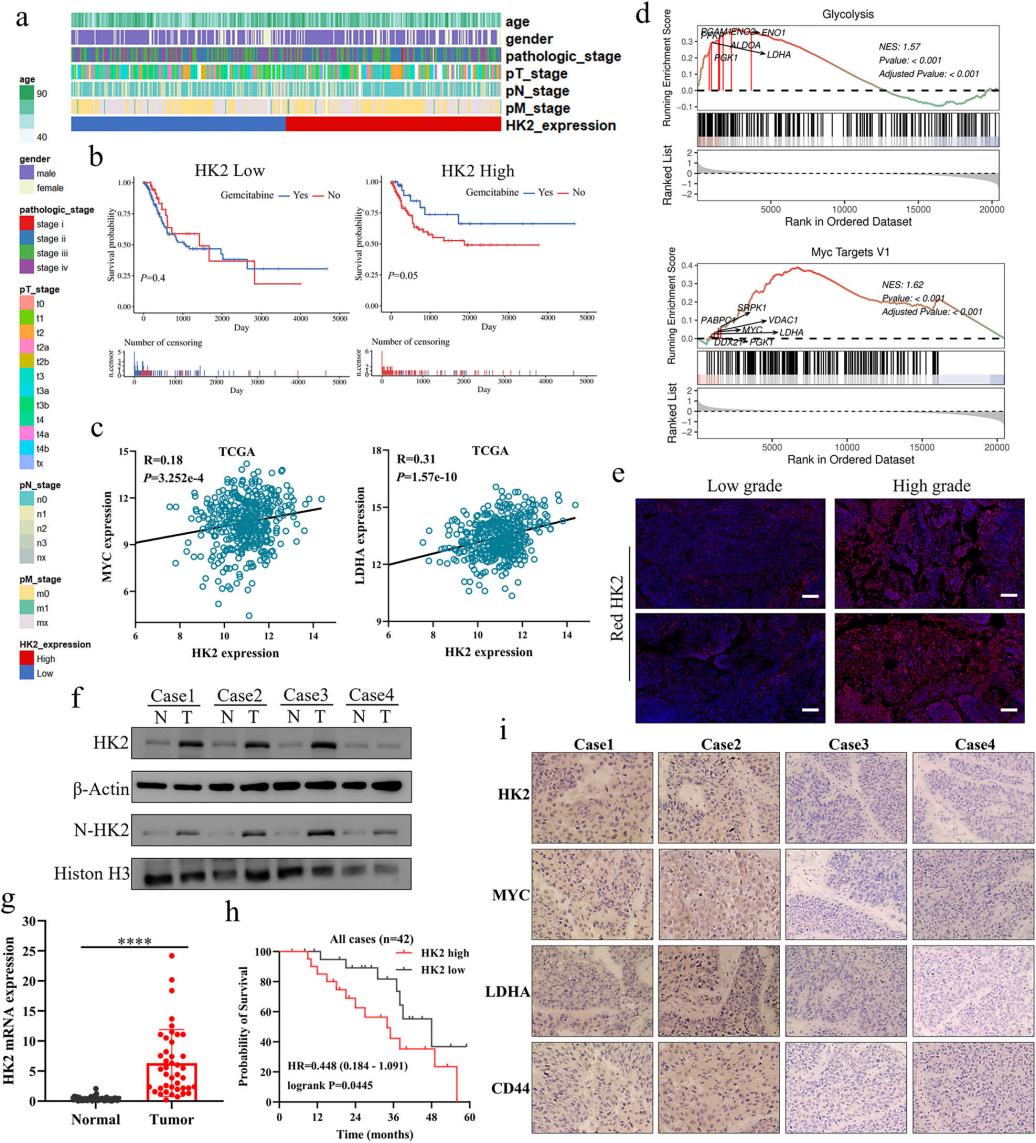
HK2 is highly expressed in bladder cancer and positively correlates with MYC. **a** Heatmap showing differences in clinicopathological features between high- and low-HK2 expression groups in the TCGA BLCA cohort. **b** Kaplan–Meier survival analysis of bladder cancer patients treated with gemcitabine, stratified by HK2 expression level in the TCGA BLCA cohort. **c** Correlation between HK2 and MYC mRNA levels in bladder cancer samples from the TCGA dataset. **d** Gene set enrichment analysis (GSEA) of glycolysis and MYC target pathways in tumors with high HK2 expression in the TCGA BLCA cohort. **e** Immunofluorescence staining of HK2 in low-grade and high-grade bladder cancer tissues. **f** Western blot analysis of total and nuclear HK2 in four paired bladder cancer tissues (T) and adjacent non-tumorous tissues (N). **g** qPCR analysis of HK2 mRNA levels in 42 paired bladder cancer and adjacent non-tumorous tissues. **h** Kaplan–Meier survival analysis based on HK2 expression levels in the cohort described in (**g**) using the median HK2 mRNA level as the cut-off. **i** Immunohistochemical staining for HK2, MYC, LDHA, and CD44 in bladder cancer tissues. Student’s t-test was used for statistical analysis. *P < 0.05, **P < 0.01 or ***P < 0.001 indicates a significant difference between the indicated groups.

To investigate the clinical relevance of HK2 in bladder cancer, we stratified TCGA BLCA cases into high- and low-HK2 expression groups. No significant association was observed between HK2 mRNA levels and tumor stage or grade (Fig. 8a). However, in patients treated with gemcitabine, high HK2 expression was associated with significantly improved survival, suggesting that tumors with elevated HK2 are more sensitive to gemcitabine-based therapy (Fig. 8b). Furthermore, HK2 expression was positively correlated with MYC and LDHA expression (Fig. 8c). Gene set enrichment analysis (GSEA) revealed that HK2 expression was strongly associated with the Glycolysis and MYC Targets pathways (Fig. 8d). Consistent with these findings, histopathological analysis of clinical bladder cancer specimens showed higher HK2 protein levels in advanced-stage tumors (Fig. 8e). Additionally, we found that HK2 expression is higher in bladder cancer patients with hyperglycemia (Supplementary Fig. 3b). To explore therapeutic relevance, we tested combination treatments in vitro. Lonidamine further reduced cell viability when combined with cisplatin and also enhanced the growth-inhibitory effect of a PD-L1 inhibitor (Supplementary Fig. 3c, d). Using Western blot analysis of paired bladder cancer and adjacent non-tumorous tissues, we found that cytoplasmic HK2, nuclear HK2, and MYC protein levels were higher in tumor tissues than in matched adjacent tissues (Fig. 8f and Supplementary Fig. 3a). Consistently, qPCR analysis showed that HK2 mRNA levels were markedly elevated in bladder cancer compared with adjacent tissues (Fig. 8g). Patients were then stratified into high- and low-HK2 expression groups, and those with high HK2 expression exhibited significantly poorer overall survival (Fig. 8h). Immunohistochemical analysis further demonstrated that HK2 expression was positively correlated with MYC, LDHA, and CD44 expression in bladder cancer tissues (Fig. 8i). Together, these findings support a role for HK2 in promoting the malignant progression of bladder cancer and reinforce its strong positive association with MYC and LDHA.

## Discussion

We identify a noncanonical nuclear function of HK2 that links hyperglycemia to MYC-driven glycolysis and stemness programs in bladder cancer. High glucose promotes HK2 nuclear accumulation, where HK2 binds MYC, co-occupies glycolytic gene promoters (HK2 and LDHA), and enhances transcription. Consistent with this mechanism, glycemic control or HK2 inhibition suppresses hyperglycemia accelerated tumor growth and HK2-MYC associated signatures in TCGA bladder cancer datasets.

Nuclear translocation of metabolic enzymes can couple metabolic cues to transcriptional programs. However, whether and how a high-glucose microenvironment shapes MYC-dependent transcriptional programs by modulating interactions with metabolic enzymes has remained poorly defined. Our work extends this paradigm by demonstrating that high glucose induces nuclear accumulation of HK2 in a dose- and time-dependent manner and that nuclear HK2 directly engages MYC through a specific binding interface. Regarding clinical relevance, 5 mM approximates normoglycemia, whereas typical diabetic hyperglycemia is in the 7–11 mM range; thus, 25–30 mM represents a severe/poorly controlled hyperglycemic condition, while 40 mM is likely supraphysiologic and closer to an extreme hyperglycemic crisis level. Accordingly, the reduction of nuclear HK2 observed at ≥40 mM glucose may reflect glucotoxic stress rather than a typical diabetic exposure. A plausible mechanism is that very high glucose induces hyperosmolar or oxidative stress that alters HK2 trafficking or accelerates turnover, thereby limiting nuclear accumulation. Beyond glucose, other microenvironmental cues such as hypoxia/HIF-1 signaling or growth factor–driven pathways (PI3K–AKT/MAPK) may also modulate HK2 nuclear trafficking, which warrants further investigation. Biophysical and biochemical assays support a high-affinity interaction between HK2 and the central region of MYC, which is stronger than that observed for HK1, and show that this complex is functionally competent to activate transcription of HK2 and LDHA. While HK1 can also interact with MYC, we prioritized HK2 because it is more prominently and glucose-dependently localized in the nucleus and shows a stronger defined interaction with MYC in our modeling. These results reveal HK2 as a MYC cofactor in the nucleus and define a previously unrecognized HK2–MYC transcriptional axis.

Our data further indicate that the nuclear HK2–MYC complex constitutes a self-amplifying circuit that couples upstream metabolic signals to downstream transcriptional programs. By binding to and activating the promoters of HK2 and LDHA, the complex reinforces its own expression and that of key glycolytic effectors, thereby sustaining a high-glycolysis state. Multiple orthogonal assays consistently showed increased glycolytic flux under high-glucose conditions and suppression upon HK2 knockdown or pharmacologic inhibition, consistent with a central role of the HK2–MYC axis in driving glycolytic reprogramming. Importantly, this metabolic rewiring is functionally linked to stemness-associated phenotypes: high glucose enhances the expression of stemness markers such as CD44, CD133, and OCT4, whereas HK2 knockdown or HK2 inhibition attenuates these effects. Partial rescue of stemness features by high glucose in HK2-deficient cells suggests that nuclear HK2 acts as an amplifier rather than the sole determinant of the stemness program. These observations provide a transcriptional explanation for the frequent co-occurrence of enhanced glycolysis and cancer stem–like properties in bladder cancer and implicate nuclear HK2 as a key mediator of this linkage.

The translational relevance of this metabolic–transcriptional coupling mechanism is supported by our in vivo and clinical data. In mouse models, diet-induced hyperglycemia accelerates the growth of bladder cancer xenografts, whereas metformin-mediated glycemic control or lonidamine-mediated HK2 inhibition suppresses tumor growth. Notably, lonidamine reduces nuclear and cytoplasmic HK2 and MYC expression in tumors without affecting systemic glucose levels, indicating that HK2 represents a druggable node downstream of hyperglycemia. At the clinical level, HK2 is upregulated in human bladder cancer tissues and positively correlates with MYC and LDHA expression. High HK2 expression is associated with enrichment of glycolysis and MYC target gene signatures and with worse overall survival, particularly in patients with hyperglycemia. Intriguingly, in our cohort, patients with high HK2 expression experienced better outcomes following gemcitabine-based chemotherapy. One possible explanation is that enhanced glycolytic flux through HK2 increases carbon flow into the pentose phosphate pathway and nucleotide biosynthesis, thereby rendering tumor cells more dependent on nucleotide production and more susceptible to DNA-targeting agents. Although this hypothesis requires direct experimental validation, our findings raise the possibility that HK2 expression and glycemic status could be exploited as biomarkers to stratify patients for metabolic interventions and chemotherapeutic regimens.

Recent MYC-centered therapeutic advances further support targeting MYC-driven programs in bladder cancer, including an intravesical AAV-delivered c-MYC–activated gene-circuit platform that addresses intratumor heterogeneity and enhances antitumor immunity [32]. Other emerging strategies, such as the ferroptosis-inducing Fe-EGCG@RSL3 nanomedicine that potentiates anti-PD-1 therapy [33] and engineered OMVs delivering CXCL9/IL12 that synergize with PD-1/PD-L1 blockade [34], highlight rational combination opportunities, for which pharmacologic disruption of the HK2–MYC axis may serve as a complementary small-molecule component.

This study has several limitations that warrant consideration. First, although diet-induced hyperglycemia recapitulates key features of a high-glucose systemic state, it may not fully model the complex metabolic and hormonal alterations present in patients with long-standing diabetes. Future studies using additional models of diabetes and hyperglycemia, as well as prospective clinical cohorts with detailed metabolic profiling, will be important to validate the generalizability of our findings. Second, lonidamine and metformin are pleiotropic agents that act on multiple targets and pathways, including mitochondrial function, AMPK signaling, and insulin sensitivity. [35, 36]. Thus, the antitumor effects observed in vivo likely reflect a combination of HK2-dependent and HK2-independent mechanisms. Given the limited specificity of lonidamine, an important translational direction is to develop agents that directly disrupt the HK2–MYC protein–protein interaction (PPI) interface. Our biochemical and modeling results provide a basis for future structure-guided discovery of small molecules or peptides that block HK2–MYC binding with improved specificity. Third, our work focuses on bladder cancer, but hyperglycemia and diabetes are also strongly linked to other malignancies. Whether the HK2–MYC axis operates in these contexts, and how it intersects with immune responses and other components of the tumor microenvironment, remain important open questions.

**Fig. 9:**
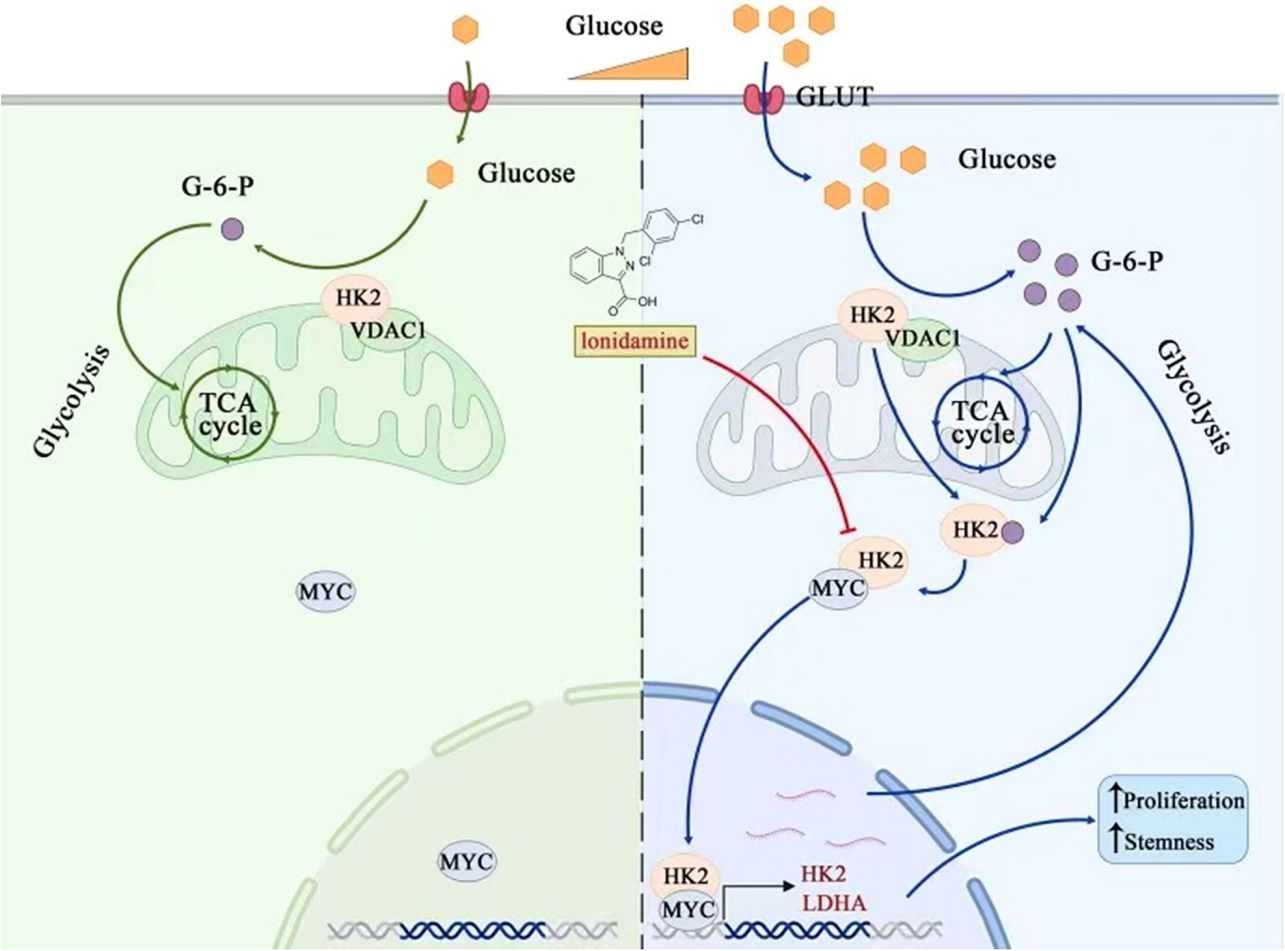
Proposed model in which nuclear HK2 associates with MYC to form a transcriptional complex that activates glycolytic gene transcription and promotes proliferation and stemness of bladder cancer cells.

In summary, our findings identify a nuclear HK2–MYC transcriptional axis as a key mechanism by which a high-glucose microenvironment drives glycolytic reprogramming and stemness in bladder cancer. By linking a common systemic comorbidity to tumor cell–intrinsic gene-expression programs, this metabolic–transcriptional coupling paradigm provides a conceptual framework for understanding how metabolic diseases can fuel cancer progression. The demonstration that HK2 is upregulated in human tumors, correlates with adverse clinical outcomes, and is amenable to pharmacologic modulation suggests that targeting HK2, in combination with glycemic control and existing chemotherapies, may represent a rational strategy for the treatment of patients with bladder cancer and coexisting hyperglycemia or diabetes. The proposed working model is summarized in Fig. 9.

## Materials and methods

### Cell lines and cell culture

Human bladder cancer cell lines UMUC3, T24, 5637, and RT4, as well as the human embryonic kidney cell line HEK293T, were obtained from the Cell Bank of the Chinese Academy of Sciences (Shanghai, China). UMUC3 and HEK293T cells were cultured in DMEM medium (HyClone, USA). T24 and 5637 cells were cultured in RPMI 1640 medium (HyClone, USA), and RT4 cells were cultured in McCoy’s 5 A medium (Gibco, USA). All media were supplemented with 10% heat-inactivated fetal bovine serum (FBS; Gibco, USA) and 1% penicillin–streptomycin. Cells were maintained at 37 °C in a humidified incubator with 5% CO₂. All cell lines were authenticated by STR profiling and were routinely tested and confirmed negative for mycoplasma contamination prior to experiments. Transient transfection of expression plasmids was performed as follows. Cells were cultured in 25 cm² flasks, and 5 µg of the indicated expression plasmid DNA was mixed with serum-free medium. Lipofectamine 3000 (Thermo Fisher Scientific, USA) was then added to the mixture and incubated for 15 min. The transfection mixture was subsequently added to the cells, which were cultured for the indicated time before subsequent experiments.

### Patients and clinical samples

From February 2022 to October 2024, 60 paired bladder cancer tissue samples (tumors and matched adjacent non-tumorous tissues) were collected at the First Hospital of China Medical University (Shenyang, China). All samples were immediately snap-frozen and stored at −80 °C until use. The study was approved by the Committee for Ethical Review of Research Involving Human Subjects of the First Hospital of China Medical University (Approval No. [2022]102), and written informed consent was obtained from all patients. All methods involving human participants were performed in accordance with the relevant guidelines and regulations.

### Western blot analysis

Whole-cell lysates were separated by SDS–PAGE and electrotransferred onto PVDF membranes. Membranes were blocked with non-fat dry milk and incubated overnight at 4 °C with primary antibodies against HK1(#2024, CST), histone H3(#4499, CST), MYC(#5605, CST), Flag tag(#8146, CST), HA tag(#3724, CST), or β-actin(#3700, CST). The membranes were then washed three times with TBS-T and incubated with the appropriate secondary antibodies for 1 h at 37 °C. After a further three washes with TBS-T, immunoreactive bands were visualized using an enhanced chemiluminescence (ECL) detection system.

### Immunofluorescence assay

Treated cells were fixed with 4% paraformaldehyde at room temperature for 10 min. Cells were then permeabilized with 0.1% Triton X-100 on ice for 10 min and blocked with fetal bovine serum (FBS) at room temperature for 1 h. Subsequently, cells were incubated overnight at 4 °C with primary antibodies against HK2(#2867, CST), MYC, or CD44. After three washes with PBS, cells were incubated with the appropriate secondary antibodies for 1 h at 37 °C. Nuclei were then counterstained with DAPI, and images were acquired using a laser-scanning confocal microscope.

### Biolayer interferometry (BLI) assay

The affinity of the HK2–MYC interaction was analyzed using an Octet K2 biolayer interferometry system, as described previously [37]. Recombinant HK2 was diluted to the indicated concentrations in Octet buffer (20 mM Tris-HCl, pH 8.0). Recombinant MYC protein bearing a hexahistidine (His₆) tag was immobilized onto nickel–nitrilotriacetic acid (Ni–NTA) biosensors. After loading, MYC-bound biosensors were washed in buffer to remove unbound protein and then transferred into wells containing recombinant HK2 to record association responses. Reference signals obtained from buffer alone and from unloaded biosensors exposed to MYC at matching concentrations were acquired as two control sets. Binding curves were corrected by double-reference subtraction, and apparent binding constants were calculated using the Octet data analysis software.

### Flow cytometry

Cells were resuspended at 1 × 10⁶ cells/mL in flow staining buffer consisting of PBS, 0.5% bovine serum albumin, 2 mM EDTA, and 0.1% sodium azide. Cells were then incubated with antibodies against HK2(#2867, CST), CD44(#3570, CST), CD133(#64326, CST), or OCT4(#2840, CST) for 1 h at 4 °C in the dark. Stained cells were analyzed using a flow cytometer.

### Immunoprecipitation and immunoblotting analysis

Proteins were extracted in RIPA buffer or 1% NP-40 lysis buffer (50 mM Tris-HCl, pH 8.0, 150 mM NaCl, 5 mM EDTA, 1% NP-40, and 10% glycerol) supplemented with protease inhibitor cocktail and PhosSTOP phosphatase inhibitor cocktail (Roche). For co-immunoprecipitation, cleared cell lysates were incubated with the indicated antibodies overnight at 4 °C and then with 20 µL Protein A/G Dynabeads (Life Technologies, Carlsbad, CA, USA) for 2 h at 4 °C. Immunocomplexes were washed, eluted in SDS sample buffer, separated by SDS–PAGE, and transferred to PVDF membranes. After blocking, membranes were incubated with the appropriate primary antibodies followed by HRP-conjugated secondary antibodies. Signals were detected by enhanced chemiluminescence (ECL).

### Molecular docking and molecular dynamics simulations

Three-dimensional structures of HK2 (AF-P52789-F1), HK1 (PDB ID: 1CZA), and MYC (AF-P01106-F1) were obtained from the Protein Data Bank and the AlphaFold Protein Structure Database. Protein–protein docking was performed using ZDOCK 3.0.2. PyMOL software was used to analyze potential amino acid binding sites at the protein–protein interfaces. Molecular dynamics simulations of the HK2–MYC complex were carried out with Amber 14. Energy minimization and molecular dynamics simulations were performed using the sander and pmemd modules implemented in Amber 14. Binding free energies were calculated using the MM/PBSA method.

### Purification of recombinant proteins

His-HK2 and GST-MYC were expressed in bacteria and purified as described previously [38]. Briefly, the corresponding constructs were expressed in Escherichia coli BL21 cells. Cultures were grown at 37 °C until the OD₆₀₀ reached 0.6, and protein expression was induced with 0.5 mM IPTG overnight. For GST-tagged proteins, cleared lysates were loaded onto a GSTrap HP column, washed with five column volumes of PBS, and eluted with 10 mM reduced glutathione. For His-tagged proteins, cleared lysates were loaded onto a Ni–NTA column, washed with five column volumes of 10 mM imidazole, and eluted with 250 mM imidazole. Proteins were then desalted and buffer-exchanged into PBS using 10 kDa molecular weight cutoff spin columns.

### GST pull-down assay

GST pull-down assays were performed as previously described [39]. Briefly, glutathione agarose beads were incubated with purified proteins overnight, washed five times with lysis buffer, and the bound proteins were eluted with 1× SDS loading buffer prior to immunoblot analysis.

### Real-time PCR

Total RNA was extracted from the indicated tissues or cells using TRIzol reagent (Invitrogen, USA) and reverse-transcribed with PrimeScript RT Master Mix (Takara, Japan). Real-time quantitative PCR (qPCR) was performed using SYBR Premix Ex Taq II (Takara). β-actin was used as an internal control. The primer sequences used in this study are listed in Supplementary Table 1.

### Extracellular acidification rate

The extracellular acidification rate (ECAR) was measured according to the manufacturer’s instructions (Seahorse Bioscience, USA). UMUC3 cells were seeded at 5000 cells per well in Seahorse microplates and exposed to the indicated treatments. Before measurement, the culture medium was removed and replaced with assay medium. Subsequently, 10 mM glucose, 5 µM oligomycin, and 50 mM 2-deoxy-D-glucose (2-DG) were sequentially injected according to the manufacturer’s protocol.

### Biochemical assays

HK activity, as well as lactate, ATP, and pyruvate levels, were measured using colorimetric assay kits (BioVision, USA) according to the manufacturer’s instructions.

### CCK-8 assay

Cells were seeded into 96-well plates at 1500 cells per well and exposed to the indicated treatments. The culture medium was then replaced with fresh medium containing Cell Counting Kit-8 (CCK-8) reagent (Sigma, USA) according to the manufacturer’s instructions. After incubation for 1 h at 37 °C, absorbance at 450 nm was measured using a microplate reader.

### ChIP assay

A chromatin immunoprecipitation (ChIP) assay kit (Millipore) was used according to the manufacturer’s instructions. In brief, cells were fixed in 1% formaldehyde for 12 min and then quenched with glycine for 5 min. After crosslinking, cells were collected, lysed in buffer containing protease inhibitors, and subjected to sonication for 4 min to shear chromatin into fragments of approximately 200–500 bp. The sonicated lysates were then incubated with anti-HK2 antibody (#2867, CST) and protein A–Sepharose beads at 4 °C; normal IgG was used as a negative control. After 24 h of incubation, the immune complexes were washed with the buffers supplied in the kit, and crosslinks were reversed by heating at 65 °C for 2 h. The recovered DNA was purified using DNA spin columns (Millipore) and analyzed by quantitative PCR (qPCR).

### Luciferase reporter assay

Promoter regions of HK2 and LDHA were cloned separately into the pRL-TK luciferase reporter vector. Reporter constructs carrying mutations in the MYC-binding site within these promoter regions were generated in parallel. The indicated cells were transfected with wild-type or mutant reporter plasmids using Lipofectamine 3000 (Thermo Fisher Scientific, USA). 48 h after transfection, cells were lysed, and luciferase activity was measured according to the manufacturer’s instructions.

### Xenograft model

All animals were cared for and treated in accordance with institutional guidelines and the protocol approved by the Animal Care and Use Committee of China Medical University (IACUC Issue No. KT2022608). All methods involving animals were performed in accordance with the relevant guidelines and regulations. Female BALB/c nude mice (4–5 weeks old, ~20 g) were randomly assigned to three groups (*n* = 5). Outcome assessment was performed blinded to group allocation. UMUC3 cells (5 × 10⁶ per mouse) were injected subcutaneously into the same groin of each mouse. To induce hyperglycemia, mice in the glucose(+) groups were fed a high-fat diet (HFD; 60% kcal from fat, 20% kcal from carbohydrate, and 20% kcal from protein), whereas glucose(−) controls received standard chow. For drug intervention, metformin was administered by oral gavage at 250 mg/kg/day, and lonidamine was administered at 50 mg/kg/day. Blood glucose was measured from the tail vein using a glucometer at baseline and every 3 days thereafter. After 24 days of observation, mice were weighed and euthanized; xenograft tumors were excised, weighed, and processed for downstream analyses. Animals with unsuccessful tumor engraftment (no measurable tumor formation) were pre-established to be excluded from analysis.

### Immunohistochemistry

Immunohistochemical staining was performed as previously described. Briefly, paraffin-embedded tissue sections were deparaffinized and rehydrated, and endogenous peroxidase activity was blocked by incubation with 3% H₂O₂. Antigen retrieval was carried out by incubating the sections with proteinase K at 37 °C for 15 min. Sections were then incubated overnight at 4 °C with primary antibodies against HK2, MYC, LDHA, or CD44 (all from Cell Signaling Technology, CST, USA). After washing, sections were incubated with the appropriate biotinylated secondary antibodies at room temperature for 1 h, followed by color development using an avidin–biotin complex (ABC) detection kit (Vector Laboratories, USA).

### Schematic diagrams

Schematic diagrams were created using FigDraw.

### RNA-seq processing

RNA sequencing (RNA-seq) data and associated clinical information for bladder urothelial carcinoma (BLCA) were downloaded from The Cancer Genome Atlas (TCGA; http://cancergenome.nih.gov/).

### Statistical analysis

All data were analyzed using GraphPad Prism v9.0. Differences between groups were evaluated using Student’s t-test in GraphPad Prism. Differences in variance between groups were assessed using Levene’s test to confirm that no extreme differences in variability were present. Data are presented as the mean ± SD of at least three independent experiments. Values of *P < 0.05, **P < 0.01, or ***P < 0.001 were considered statistically significant.

## Supplementary information

Supplementary Table 1

Supplementary Fig.s

WB original

## Data Availability

The data that support the findings of this study are available on request from the corresponding author. The data are not publicly available due to privacy or ethical restrictions.
