# Supplementary material for: Nuclear hexokinase 2 couples hyperglycemia to MYC-driven glycolytic and stemness programs in bladder cancer
Source: Cell Death Dis. 2026 Apr 8;17(1):493. doi: 10.1038/s41419-026-08714-0 (PMC13187003; doi:10.1038/s41419-026-08714-0)
Supplement: Supplementary file 1 — Supplementary Table 1 [file 41419_2026_8714_MOESM1_ESM.doc]

**Supplementary Table 1. qPCR primer sequences**

| Gene name | Forward Primer | Reverse Primer |
| --- | --- | --- |
| SLC2A1 | GCTAGCATGGAGCCCACCAGCAAG | AAGCTTTCACACTTGGGAATCAGCTCC |
| SLC2A4 | CTGGACCCATCCCGTGGTTCAT | CAAATGTCCGCCCTCTGGATTCAG |
| HK2 | GAGCCACCACTCACCCTACT | CCAGGCATTCGGCAATGTG |
| GPI | GGAAGGGGTACACAGGCAAG | TGGAGAAACCACTCCTTCGC |
| PFKL | GCTGCAAGGCCTTTACCACC | CCAGCCTCTCACACATGAAGT |
| ALDOA | AGATCCTCCCTGATGGGGAC | CTTCTGAGTGCAAGCATGGC |
| GAPDH | AATGGATTTGGACGCATTGGT | TTTGCACTGGTACGTGTTGAT |
| PGK1 | CCCACAGCTCCATGGTAGGA | TTGGCCAGTCTTGGCATTCT |
| PGAM1 | TCGCTCTCTTCTGCACTGAG | ACCTGGAGAACCGCTTCAG |
| ENO1 | GCCTCCTGCTCAAAGTCAAC | AACGATGAGACACCATGACG |
| ENO2 | GAAGCCATCCAAGCGTGCAA | AAAGTGCGGAACCCCAATGA |
| PKM2 | AGAACTTGTGCGAGCCTCAA | GGCCTTGCCAACATTCATGG |
| LDHA | ACGTGCATTCCCGATTCCTT | GGAAAAGGCTGCCATGTTGG |
| CD44 | GACACATATTGCTTCAATCTGC | GATGCCAAGATGATCAGCCATT |
| CD133 | AGTCGGAAACTGGCAGATAGC | GGTAGTGTTGTACTGGGCCAAT |
| OCT4 | CTGGGTTGATCCTCGGACCT | CCATCGGAGTTGCTCTCCA |
| ALDH1A1 | GGGCTACCTGTCCTCTTCAC | CAGGACCATCTCGTTCACTC |
| NANOG | CAGCCCCGATTCTTCCACCAGTCCC | CGGAAGATTCCCAGTCGGGTTCACC |
