# Supplementary material for: Nuclear hexokinase 2 couples hyperglycemia to MYC-driven glycolytic and stemness programs in bladder cancer
Source: Cell Death Dis. 2026 Apr 8;17(1):493. doi: 10.1038/s41419-026-08714-0 (PMC13187003; doi:10.1038/s41419-026-08714-0)
Supplement: Supplementary file 2 — Supplementary Fig.s [file 41419_2026_8714_MOESM2_ESM.docx]

**Supplement Figure Legends**

**
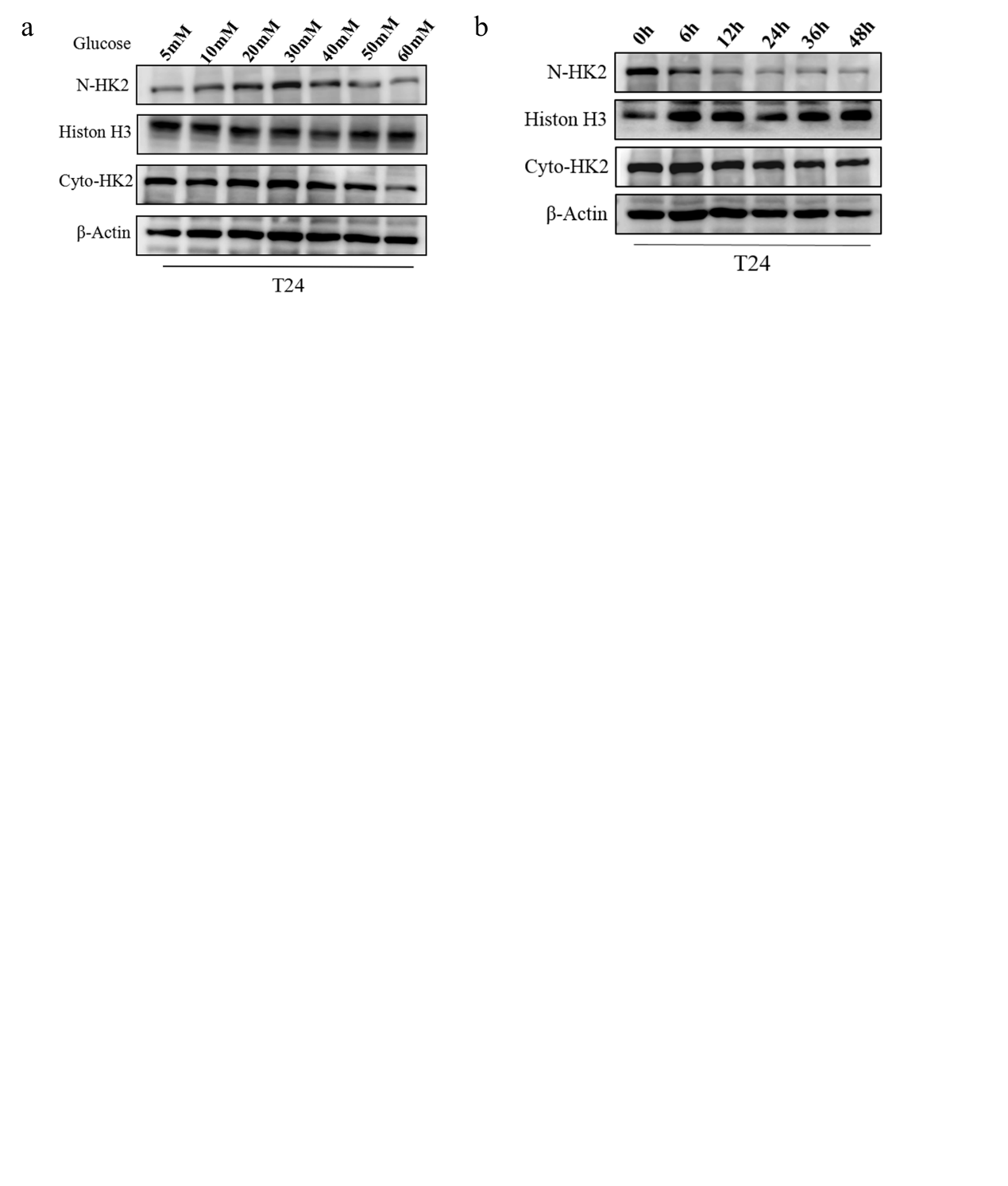
**

**Supplementary Figure S1. High glucose promotes nuclear localization of HK2 in bladder cancer cells.** (a) UMUC3 cells were treated with increasing concentrations of glucose, and nuclear and cytoplasmic HK2 expression was analyzed by Western blotting. (b) UMUC3 cells were exposed to a fixed concentration of glucose for the indicated times, and nuclear and cytoplasmic HK2 expression was analyzed by Western blotting.

**
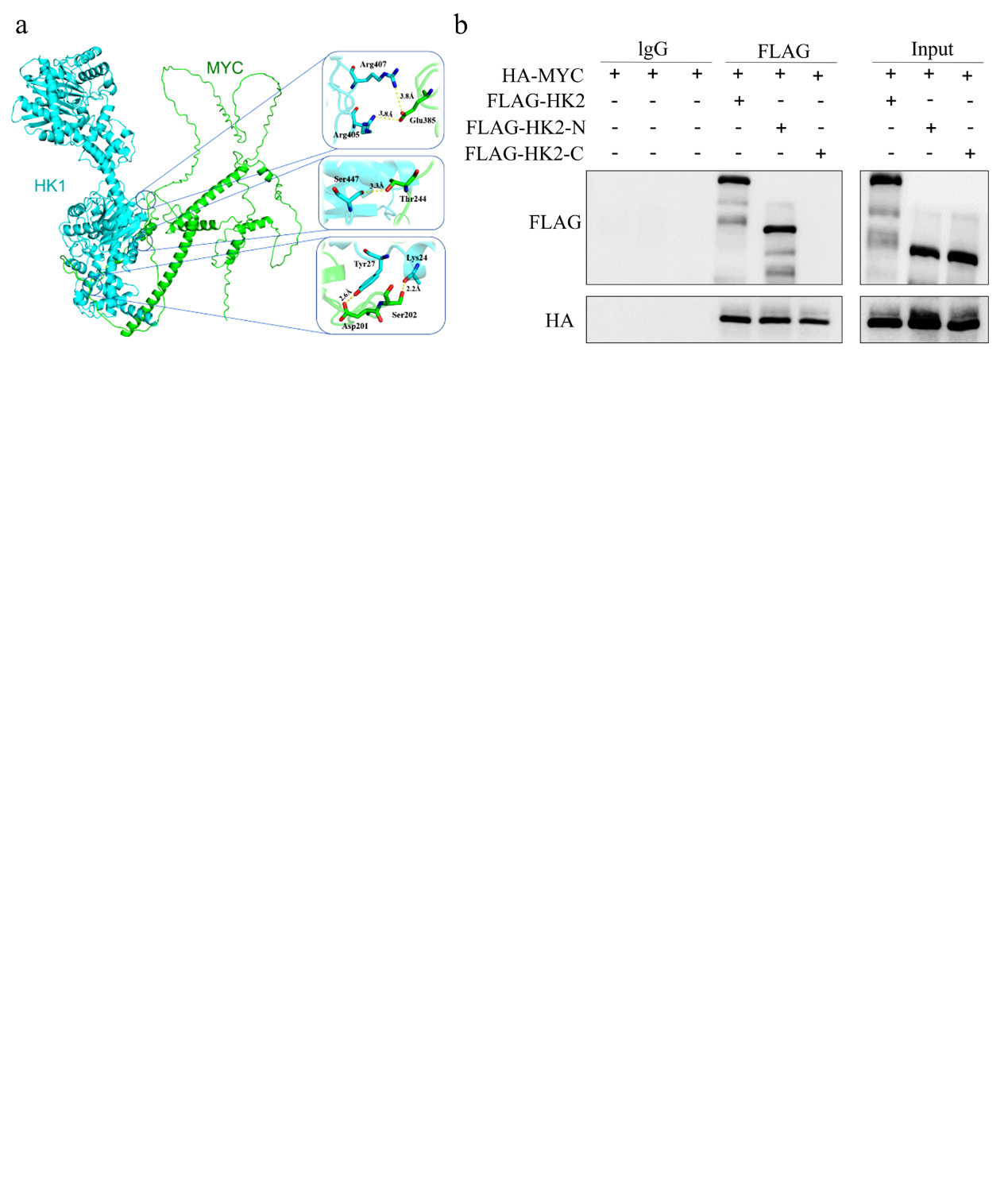
**

**Supplementary Figure S2. In vitro evidence for direct binding between HK2 and MYC.**(a) Protein–protein docking between HK1 and MYC was performed using ZDOCK.(b) Co-immunoprecipitation (Co-IP) analysis of interactions between MYC and distinct HK2 domains.


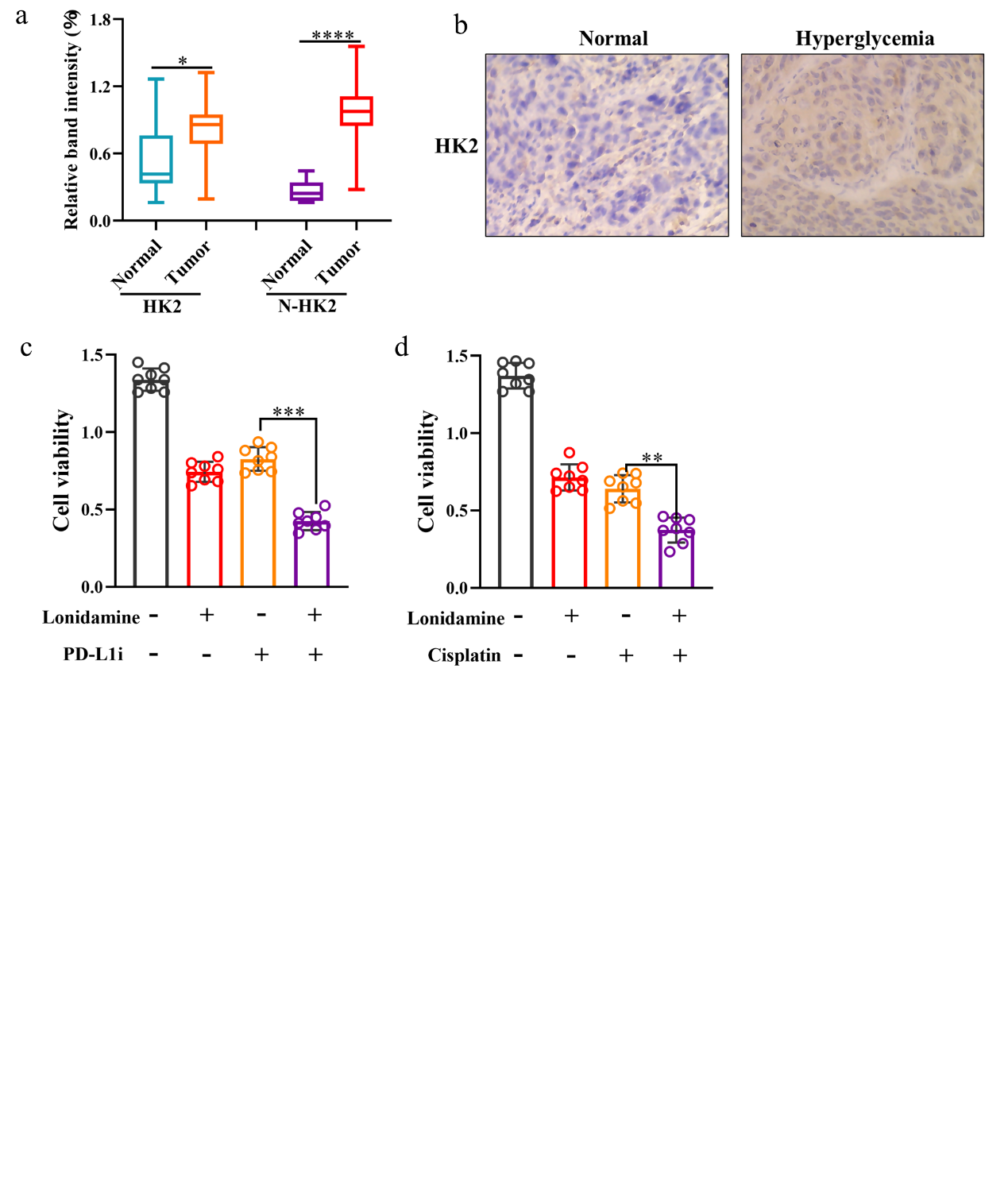


**Supplementary Figure S3. HK2 protein is highly expressed in bladder cancer tissues and HK2 inhibition enhances the anti-tumor effects of cisplatin and anti–PD-L1 antibody in vitro.**(a) Western blot analysis of total and nuclear HK2 in four pairs of bladder cancer and adjacent non-tumorous tissues. (b) Immunohistochemical staining of HK2 in bladder cancer specimens from patients with normal and hyperglycemia. (c) Cell viability after treatment with lonidamine or PD-L1 inhibitor. (d) Cell viability after treatment with lonidamine or cisplatin.
