# Supplementary material for: Nuclear hexokinase 2 couples hyperglycemia to MYC-driven glycolytic and stemness programs in bladder cancer
Source: Cell Death Dis. 2026 Apr 8;17(1):493. doi: 10.1038/s41419-026-08714-0 (PMC13187003; doi:10.1038/s41419-026-08714-0)

figure1a

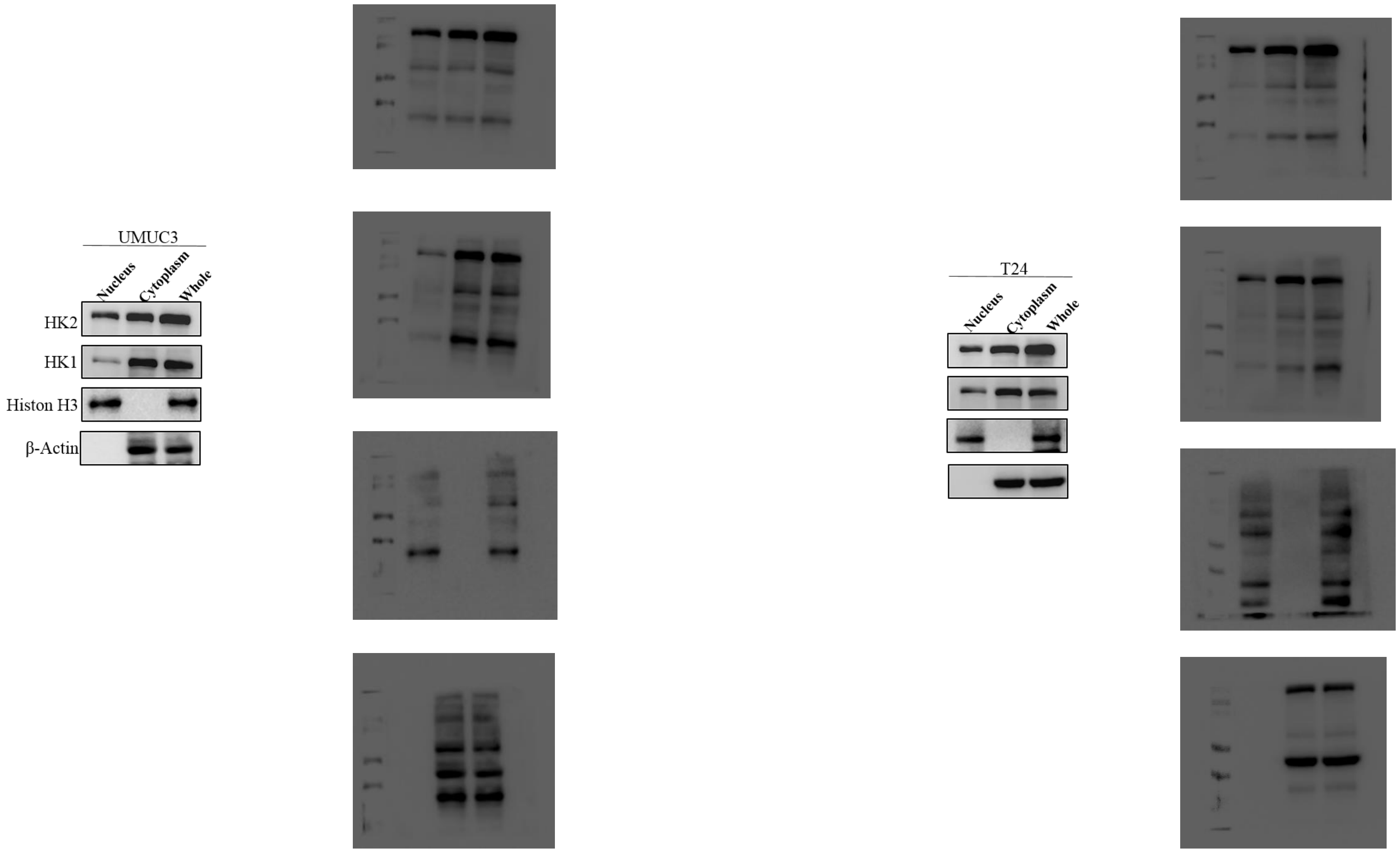
UMUC3
Nucleus
Cytoplasm
Whole
HK2
HK1
Histon H3
β-Actin
T24
Nucleus
Cytoplasm
Whole

figure1a

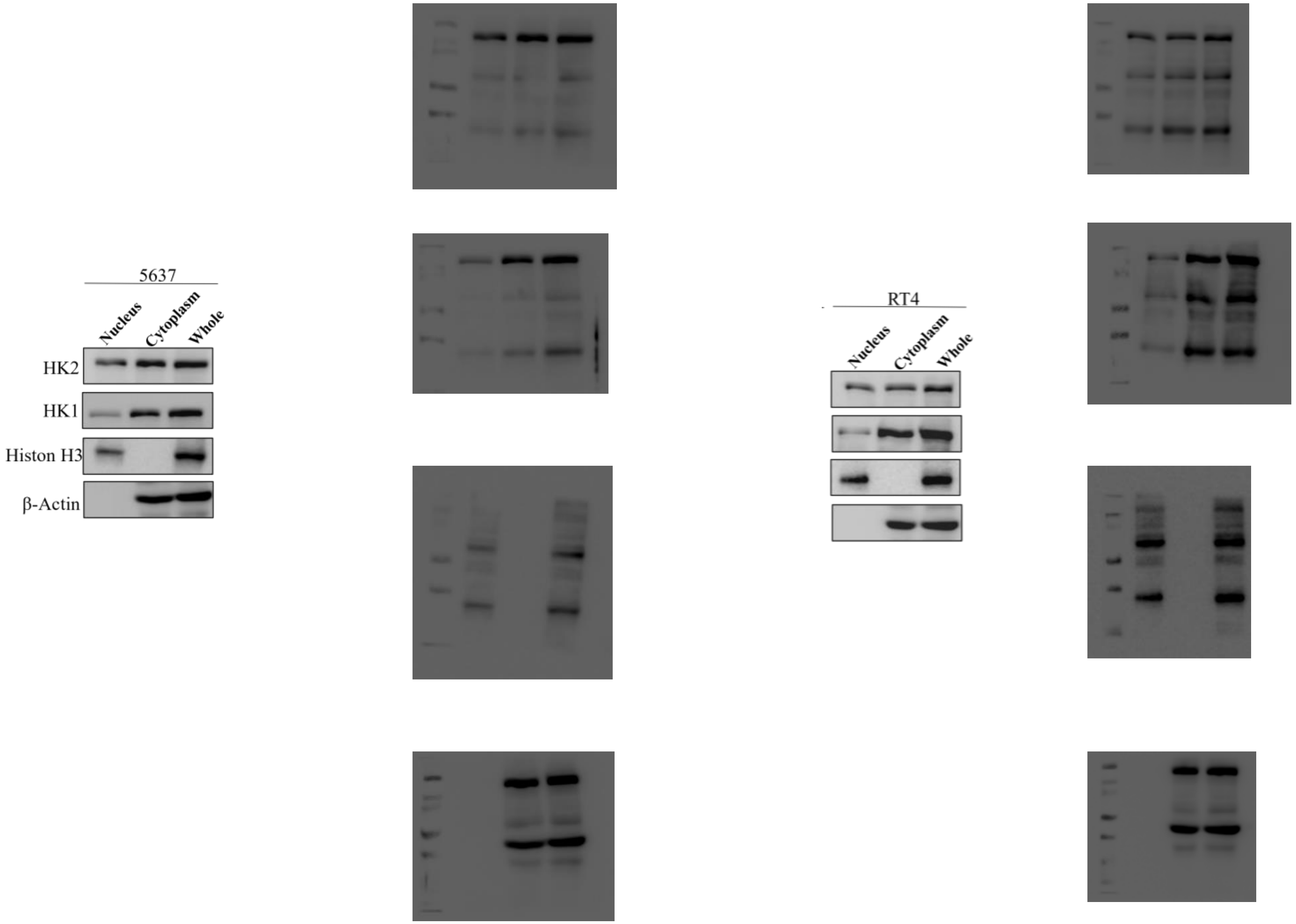
5637
Nucleus
Cytoplasm
Whole
HK2
HK1
Histon H3
β-Actin
RT4
Nucleus
Cytoplasm
Whole

figure1b

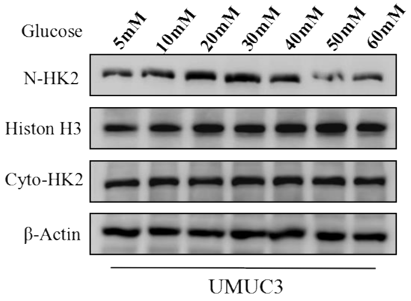
Glucose
5mM
10mM
20mM
30mM
40mM
50mM
60mM
N-HK2
Histon H3
Cyto-HK2
β-Actin
UMUC3

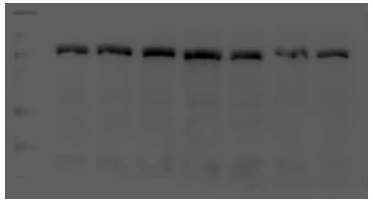

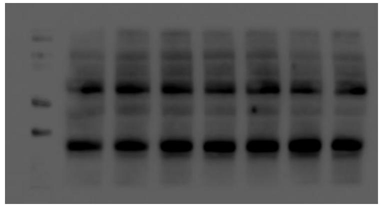

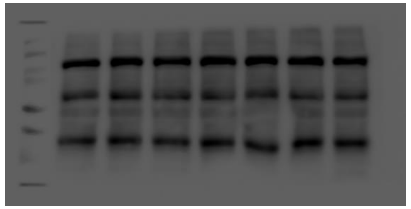

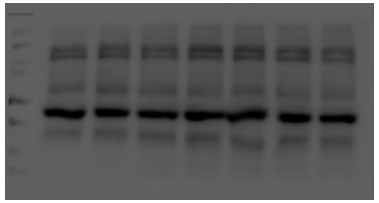

figure1c

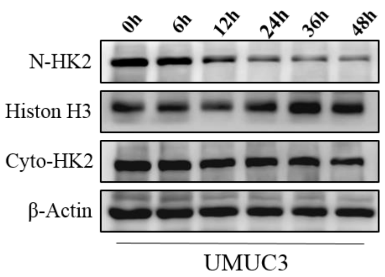

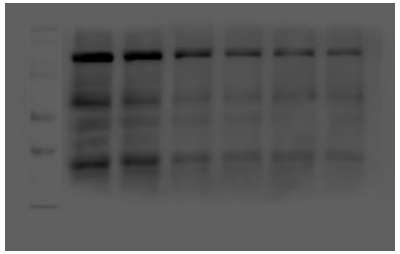

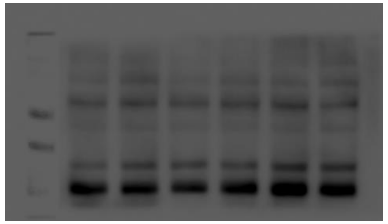

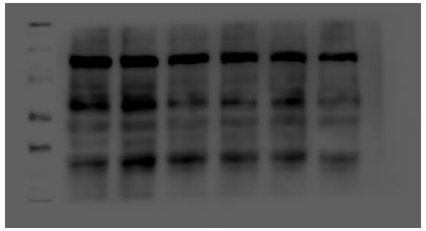

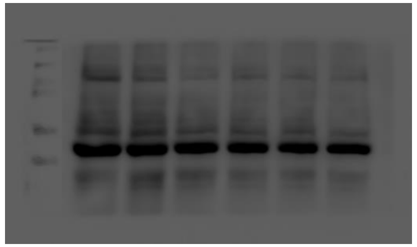

figure2c

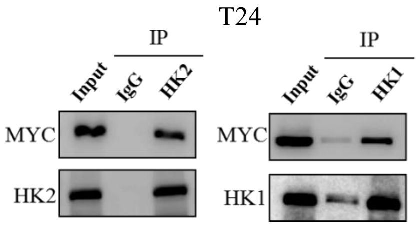
T24
IP
Input
IgG
HK2
IP
Input
IgG
HK1
MYC
HK2
MYC
HK1

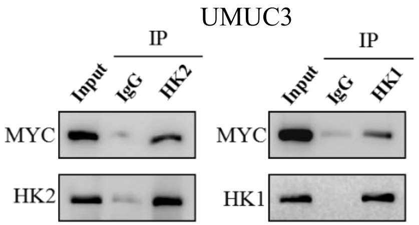
UMUC3
IP
Input
IgG
HK2
IP
Input
IgG
HK1
MYC
HK2
MYC
HK1

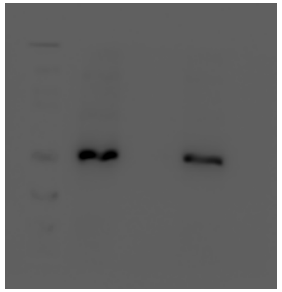

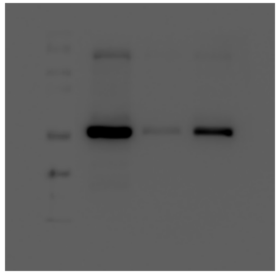

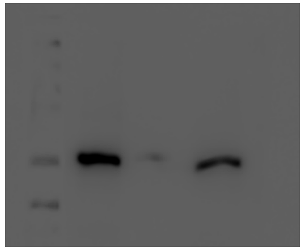

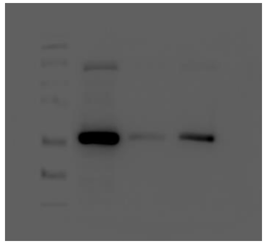

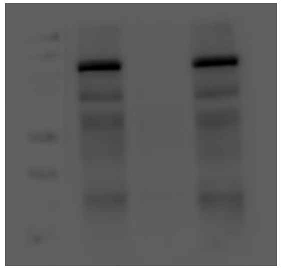

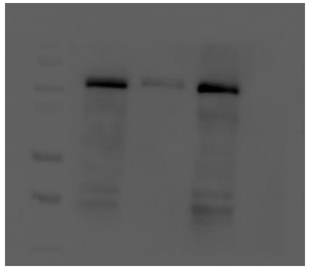

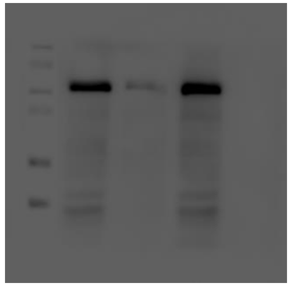

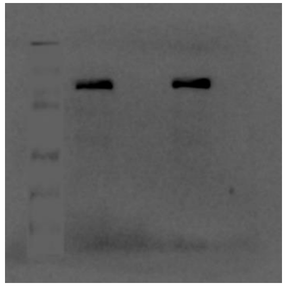

figure2d

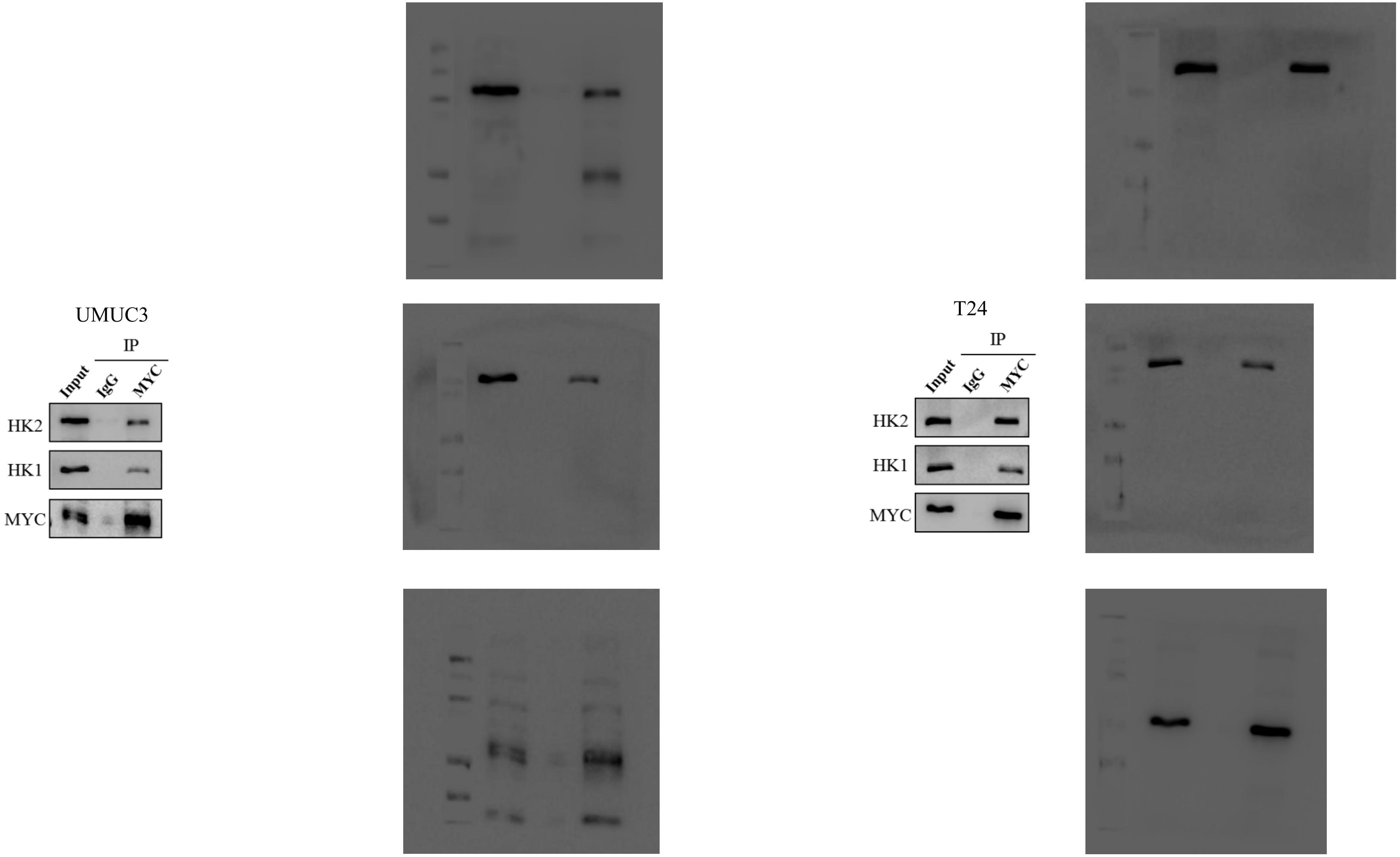
UMUC3
IP
Input
IgG
MYC
HK2
HK1
MYC
T24
IP
Input
IgG
MYC
HK2
HK1
MYC

figure2e

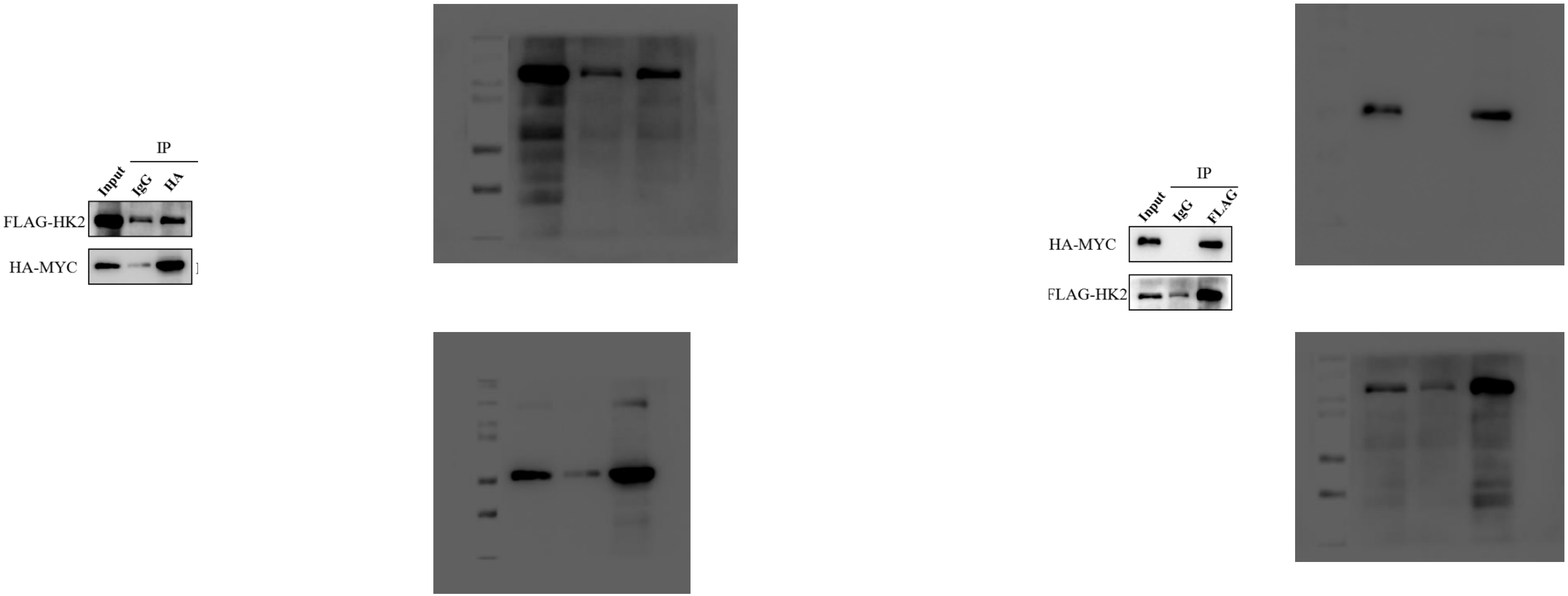
IP
Input
IgG
HA
FLAG-HK2
HA-MYC
IP
Input
IgG
FLAG
HA-MYC
FLAG-HK2

figure5g

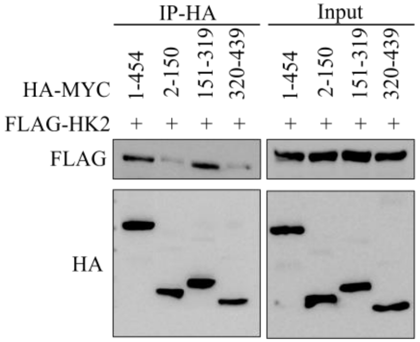
IP-HA
Input
HA-MYC
1-454
2-150
151-319
320-439
1-454
2-150
151-319
320-439
FLAG-HK2
+ + + + + + + +
FLAG
HA

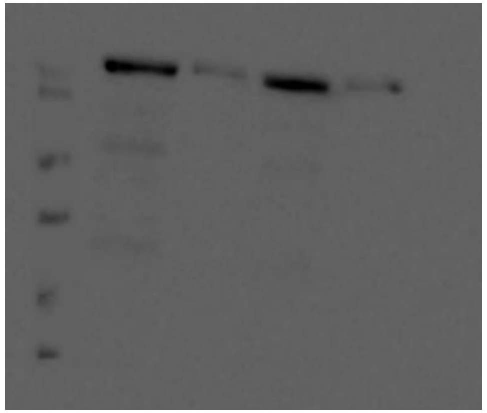

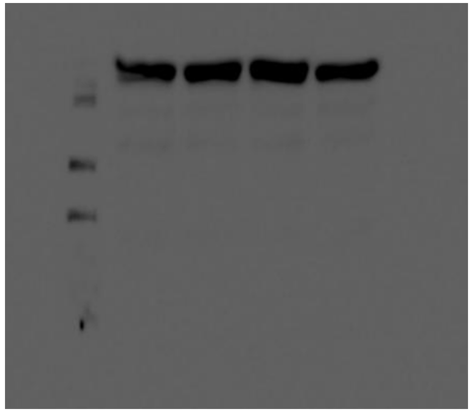

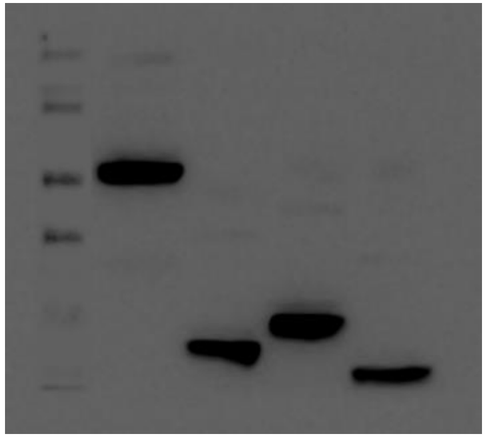

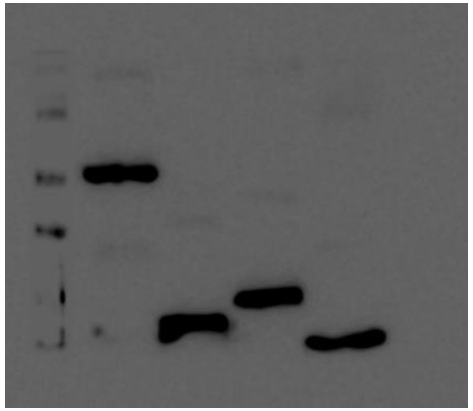

figure5h

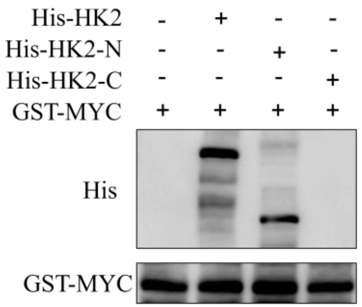

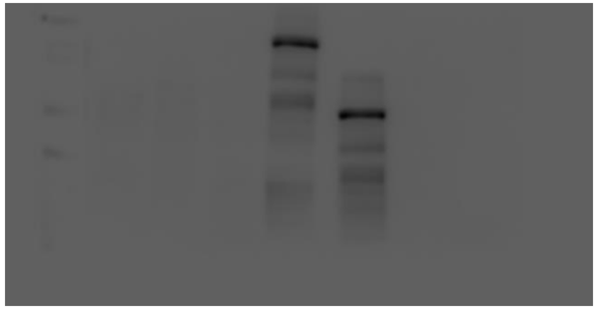

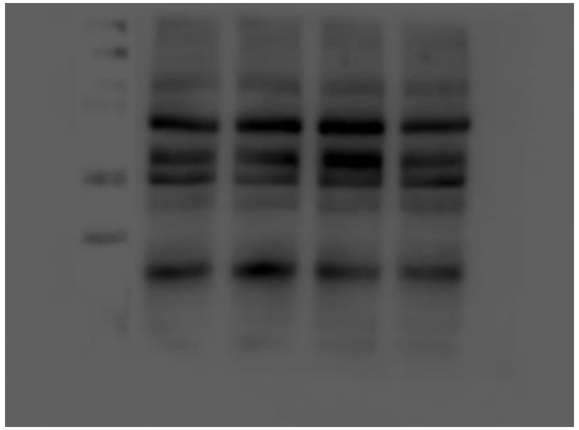

figure7g

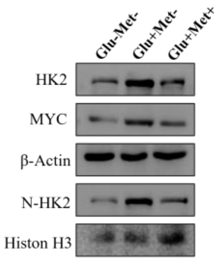
Glu-Met-
Glu+Met-
Glu+Met+
HK2
MYC
β-Actin
N-HK2
Histon H3

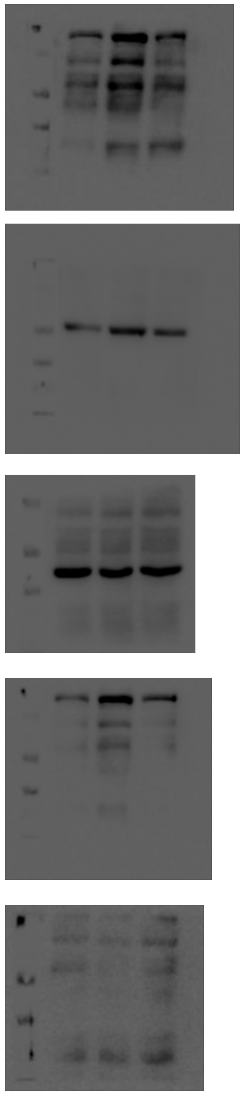

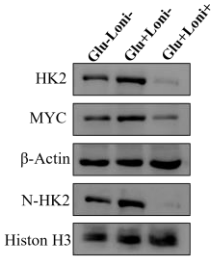
Glu-Loni-
Glu+Loni-
Glu+Loni+
HK2
MYC
β-Actin
N-HK2
Histon H3

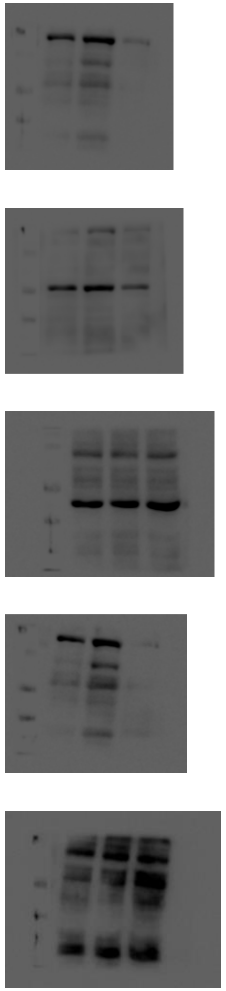

figure8f

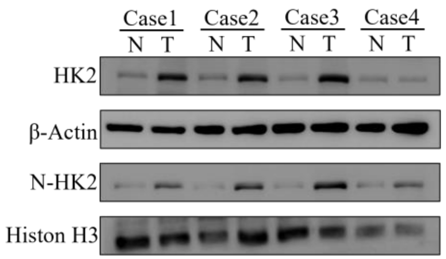

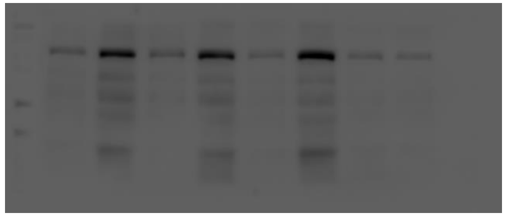

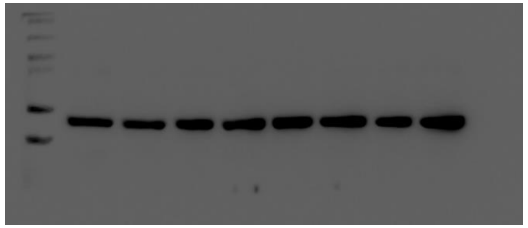

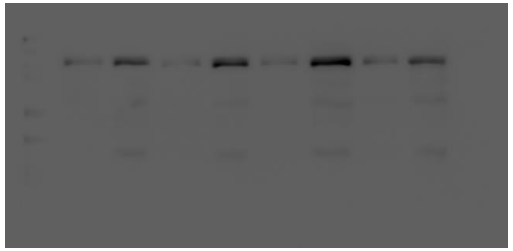

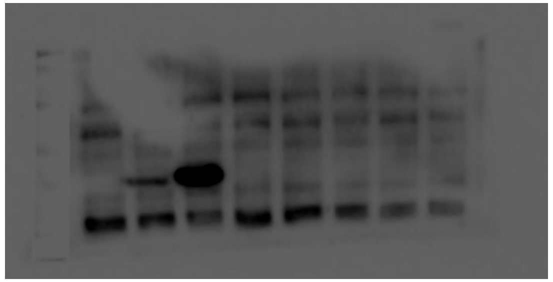

figure s1a

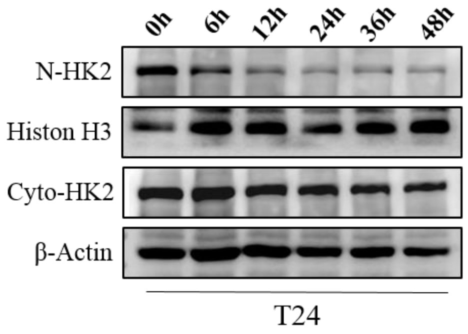

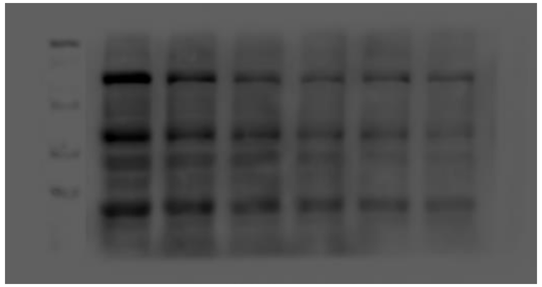

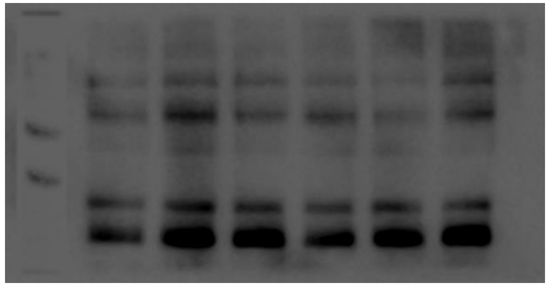

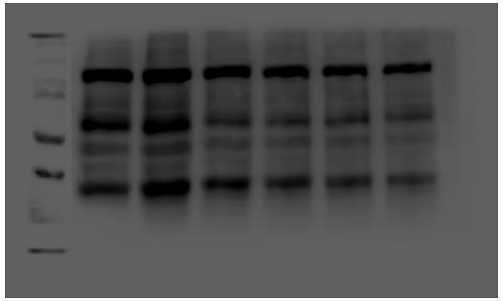

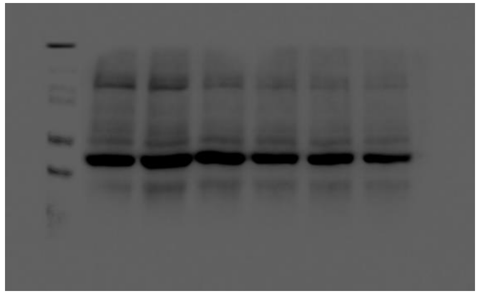

figure s1b

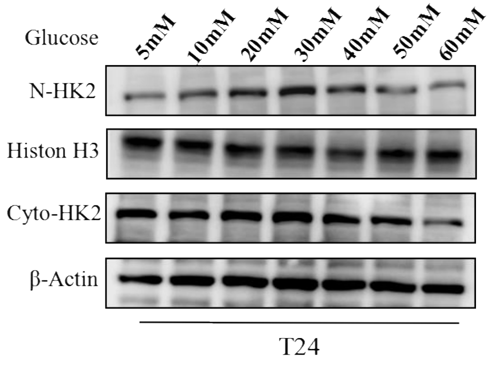
Glucose
5mM
10mM
20mM
30mM
40mM
50mM
60mM
N-HK2
Histon H3
Cyto-HK2
β-Actin
T24

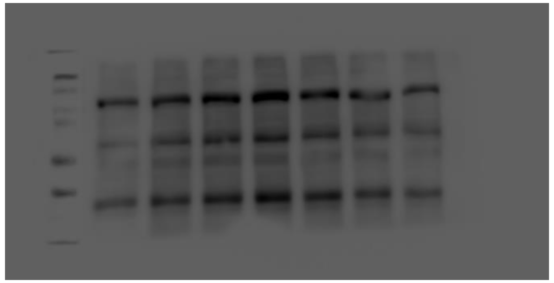

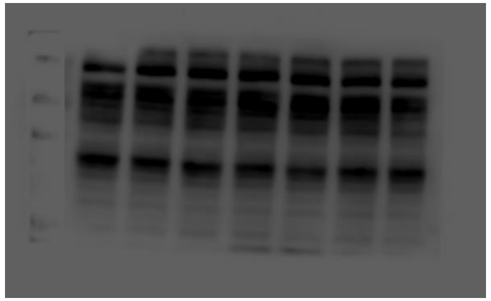

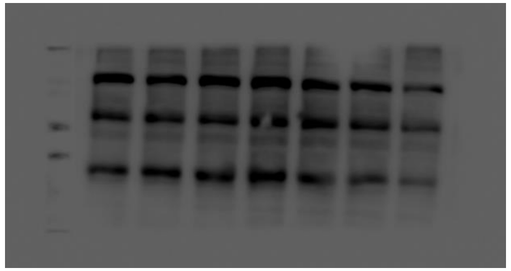

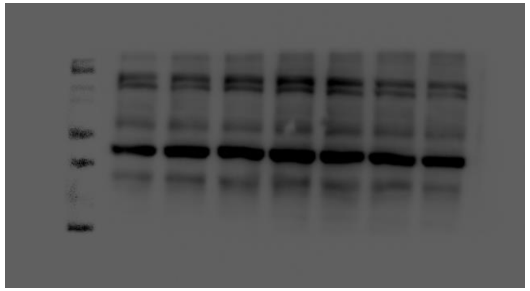

figure s2b

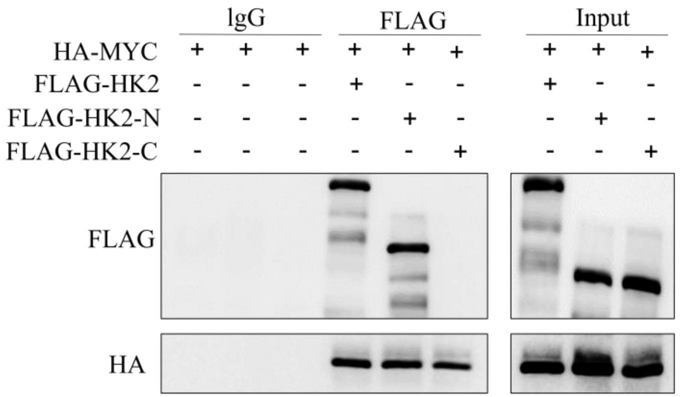

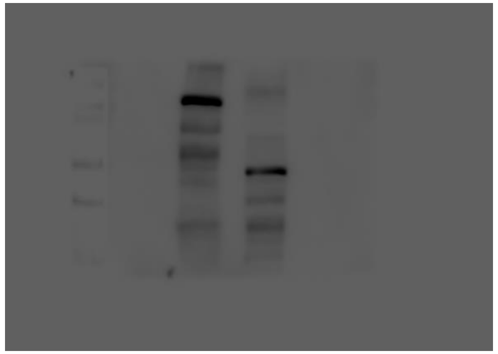

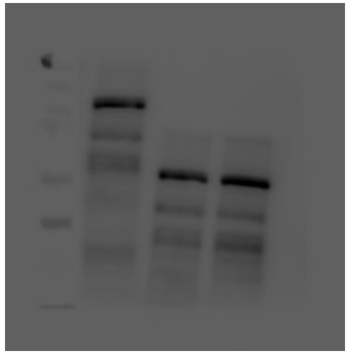

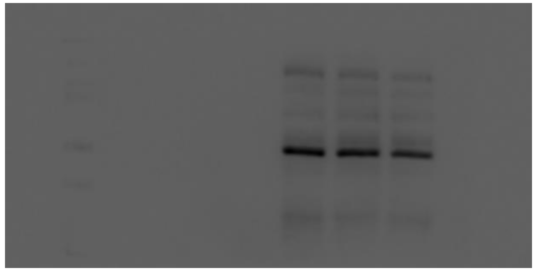

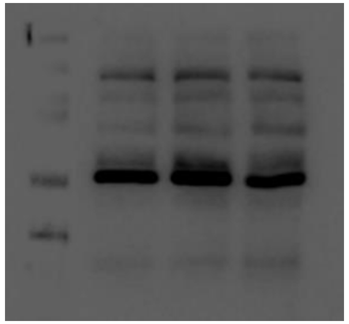

Supplement: Supplementary file 3 — WB original [file 41419_2026_8714_MOESM3_ESM.pdf]
